# Elevated CO_2_ and nitrate levels increase wheat root-associated bacterial abundance and impact rhizosphere microbial community composition and function

**DOI:** 10.1038/s41396-020-00831-8

**Published:** 2020-11-18

**Authors:** Alla Usyskin-Tonne, Yitzhak Hadar, Uri Yermiyahu, Dror Minz

**Affiliations:** 1 Soil, Water and Environmental Sciences, Agricultural Research Organization, Volcani Center, Rishon LeZion, Israel; 2 Robert H. Smith Faculty of Agriculture, Food and Environment, The Hebrew University of Jerusalem, Rehovot, Israel; 3 Soil, Water and Environmental Sciences, Agricultural Research Organization, Gilat Research Center, Negev, Israel

**Keywords:** Microbiome, Microbial ecology, Metagenomics, Microbial ecology

## Abstract

Elevated CO_2_ stimulates plant growth and affects quantity and composition of root exudates, followed by response of its microbiome. Three scenarios representing nitrate fertilization regimes: limited (30 ppm), moderate (70 ppm) and excess nitrate (100 ppm) were compared under ambient and elevated CO_2_ (eCO_2_, 850 ppm) to elucidate their combined effects on root-surface**-**associated bacterial community abundance, structure and function. Wheat root-surface-associated microbiome structure and function, as well as soil and plant properties, were highly influenced by interactions between CO_2_ and nitrate levels. Relative abundance of total bacteria per plant increased at eCO_2_ under excess nitrate. Elevated CO_2_ significantly influenced the abundance of genes encoding enzymes, transporters and secretion systems. *Proteobacteria*, the largest taxonomic group in wheat roots (~ 75%), is the most influenced group by eCO_2_ under all nitrate levels. *Rhizobiales, Burkholderiales* and *Pseudomonadales* are responsible for most of these functional changes. A correlation was observed among the five gene-groups whose abundance was significantly changed (secretion systems, particularly type VI secretion system, biofilm formation, pyruvate, fructose and mannose metabolism). These changes in bacterial abundance and gene functions may be the result of alteration in root exudation at eCO_2_, leading to changes in bacteria colonization patterns and influencing their fitness and proliferation.

## Introduction

The atmospheric concentration of carbon dioxide (CO_2_) has been increasing since the industrial revolution due to fossil-fuel burning. With the rapid increase in industrialization, CO_2_ became a major greenhouse gas, and it is expected to increase from the current ambient level (aCO_2_) of ca. 400 ppm to 800–1000 ppm, or more, at the end of 21st century [1]. In addition to its many negative effects, elevated CO_2_ (eCO_2_) is known to improve the growth of agriculturally important plants [2], and promote production of root exudates [3], which can result in increased soil microbial biomass and subsequently, increased soil microbial nitrogen demand [4, 5]. The outcome of this may be enhanced competition in the root zone between soil microorganisms and plants for available nitrogen [6], leading to progressive nitrogen limitation for the plants [7, 8]. Indeed, plant nitrogen concentration generally decreases under eCO_2_ [9, 10], especially in C3 plants such as wheat [11]. Another possible explanation for this phenomenon is that eCO_2_ inhibits nitrate-N assimilation into proteins in the shoots of C3 plants [11–13]. Thus, with the predicted increase in atmospheric CO_2_ level, an increase in the supply of nitrogen will be needed to sustain wheat growth and satisfy soil microbial nitrogen demand [14].

Elevated CO_2_ affects the soil microbial community indirectly via plant metabolism and root secretions, particularly in C3 plants [15], an effect that is locally restricted to the apical root zone [6]. Not much is known about the effect of eCO_2_ on the structure, and especially function of the rhizosphere and root-surface-associated microbial communities. In fact, the studies published so far have focused on the structure of rhizosphere microbial communities and not on root-surface-associated communities [16–18], that are likely to be even more affected by eCO_2_ and increased nitrate supply [19]. Moreover, an increase in root exudates by eCO_2_ can stimulate microbial activity in the root system and thus increase the consumption of oxygen [20], creating anoxic niches along the root profile that can promote denitrification. Denitrification is essentially an anaerobic respiration pathway in which the oxidized form of nitrogen is used as a terminal electron acceptor in the absence of oxygen. It has been previously shown that with the addition of nitrogen supply, the activity and abundance of denitrifiers having *nirS*, *nirK* and *nosZ* genes are significantly affected in the wheat rhizosphere [21]. Moreover, with the addition of nitrogen, there are alterations in the quantity and composition of wheat exudates that may have a significant impact on root microbial community structure [22]. It was previously shown that the relative abundance of *Proteobacteria*, *Actinobacteria* and *Bacteroidetes*, which often dominate the roots [23, 24], is highly influenced by nitrogen supply [22] and CO_2_ level [25].

In this study, we have focused on the root-surface-associated bacterial community, which is the one in intimate interaction with the roots. It is hypothesized that they would be the first in responding to changes in the root system. Here we examined the combined effect of eCO_2_ and nitrate levels on the structure and function of the root-surface-associated (i.e., bacteria attached to root surface) bacterial community structure and functions based on metagenome extracted from the background of host-plant (wheat) genomic data. Three possible nitrate fertilization scenarios to sustain future wheat growth were studied: (1) limited (e.g., 30 ppm nitrate), moderate (e.g., 70 ppm nitrate) and excess nitrate (e.g., 100 ppm nitrate). We anticipated that changes in function of the root-surface-associated bacterial community over a period of weeks might react to and indicate the conditions developing in the root system, as a result of changing levels of atmospheric CO_2_ and nitrate supply. Hence, by observing changes in gene functions of the root-surface-associated microbiome, we sought to reveal possible conditions to which the roots will be exposed in the future, information that might enable accommodation of wheat growth to future environmental conditions.

## Materials and methods

### Greenhouse experiments and sampling

Wheat (*Triticum turgidum* cv. Negev) was cultivated in sandy loam soil (19% clay, 6% silt, 75% sand) classified as Calcic Haploxerept. The soil was obtained from intensive agriculture field located in Eshkol region, Israel (31.248,949, 34.379,872). Potatoes, wheat and peanuts were previously grown in this field. Initial soil parameters were: pH 8.78 ± 0.04, electrical conductivity 99 ± 1 (µS/m), NO_3_-N 0.22 ± 0.02 (mg/kg), NH_4_ 0.30 ± 0.01 (mg/kg), P-PO_4_ 0.09 ± 0.01(mg/kg), total soluble organic carbon 4.0 ± 0.04 (mg/kg) and total soluble nitrogen 0.70 ± 0.02 (mg/kg).

The plants were grown for 6 weeks (from December 2016 to February 2017) as described previously [25]. Briefly, 750 g of soil was distributed in a 700-mL plastic pot, with four seeds per pot. Those pots were able to sustain up to four wheat plants for six weeks under the experimental conditions. The wheat was grown in a greenhouse with two closed-system chambers at day/night temperatures of 25 °C/18 °C ± 1 °C, and with an automatically adjusted CO_2_-supply system (Emproco Ltd., Ashkelon, Israel). The photoperiod was 9 h and the daily light integral was 12.5 MJ/day. Wheat plants were grown in a sequence of three independent experimental cycles of 6 weeks each (five pots per treatment per cycle), with a 1-week shift between cycles. Plants were grown under either ambient (400 ppm) or elevated (850 ppm) atmospheric CO_2_ levels. Nutrient solution was prepared with 90% nitrogen supplied as nitrate and 10% supplied as ammonium using KNO_3_ and NH_4_NO_3_ to provide final concentrations of 30, 70 and 100 ppm nitrate [26]. Other macronutrients were supplied in each treatment at the following rate: P-15 ppm, K-150 ppm, Mg-24 ppm, Ca-120 ppm and S-40 ppm provide by NH_4_NO_3_, KNO_3_, CaCl_2_, KCl, MgCl_2_ and KH_2_PO_4_ salts. 40 ppm S and Ca were present in the tap water. Micronutrients were supplied at a rate of 1.3 ppm Fe, 0.7 ppm Mn, 0.3 ppm Zn, 0.05 ppm Cu, and 0.0375 ppm Mo using Korotin (Haifa Chemicals, Israel), a commercial micronutrient mix. Each pot was irrigated with 50 mL of the nutrient solution four times a week. The total amount of nitrogen in the 30 ppm nitrate treatment was 36 mg/pot (equivalent of ca. 73 kg N/ha), 70 ppm nitrate treatment was 84 mg/pot (equivalent of ca. 170 kg N/ha) and in the 100 ppm treatment, 120 mg/pot (equivalent of ca. 250 kg N/ha).

### Soil and plant analyses

At the end of the 6^th^ week of growth, 15 pots (5 pots per cycle) from each treatment were sampled for soil, shoots and roots, and the following parameters were measured: soil nitrate and ammonia content, soil EC and soil pH, shoot and root dry biomass, nitrogen concentration and content in shoot and roots. Soil properties and relevant methods were as described previously [25]. Briefly, soil EC and pH were determined in a solution of 1:5 air dry sieved soil:distilled water (w/v). Nitrate and ammonium concentrations were determined using an autoanalyzer (Lachat Instruments, Milwaukee, WI or Gallery Plus, Thermo Fisher Scientific, Waltham, MA, USA). Sampled shoots and roots were dried at 60 °C for 48 h, ground and weighed to obtain dry biomass. Total nitrogen concentration was determined using an autoanalyzer (Lachat Instruments or Gallery Plus) following digestion with sulfuric acid and peroxide [27].

### Root DNA extraction for sequencing and qPCR

At the end of the 6th week of wheat growth, pots were randomly selected for DNA extraction. To obtain the root-surface-associated microbiome, wheat roots were collected in triplicate from each of the three cycles and were vortexed three time with 85% saline solution, until no visible soil particles were attached to the roots. Total DNA was extracted from 0.4 g of complete root system, using the Exgene Soil DNA mini isolation kit (GeneAll, Seoul, Korea) according to the manufacturer’s instructions.

### Generation of qPCR plasmid standards

Plasmids containing the 16S rRNA gene were generated as described previously [28, 29]. Each PCR amplification product was ligated into pGEM-T Easy Vector (Promega, Madison, WI, USA) and plasmids were transformed into BioSuper *Escherichia coli* DH5α competent cells (Bio-Lab, Jerusalem, Israel). Circular plasmid DNAs were used as the standards to create calibration curves at 10-fold dilutions for gene quantification by real-time qPCR.

### Assessment of gene copy numbers by qPCR

Copy numbers of the total bacterial community (16S rRNA gene) and translation elongation factor 1 (*TEF*, a plant housekeeping gene) were assessed using selected primers (Table S1) in roots of 6-week-old wheat plants with the StepOnePlus Real-Time PCR System (Applied Biosystems, Foster City, CA, USA). Triplicates from whole genomic DNA were diluted to 6 ng/µL and 1 µL was used in a 20-µL final reaction volume together with 50 µM forward and reverse primers and 10 µL 1X FAST MasterMix (Thermo Fisher Scientific). Three biological and three technical replicates were analyzed for each root DNA sample. Reaction efficiency was monitored in each run by means of an internal standard curve (constructed plasmids) using duplicates of 10-fold dilutions of standards ranging from 10^8^–10^2^ copies per reaction. Efficiency was 89–98% for all target genes and runs, and R^2^ values were greater than 0.99. Copy numbers of the target genes were calculated based on the relative calibration curve of the plasmid copy numbers. All data analyses were conducted using StepOne software v2.3 (Applied Biosystems).

### Shotgun sequencing

Root DNA was extracted from each of the biological triplicates, in each of the three cycles. For sequencing, the DNA of the triplicates was combined, resulting in three biological replicates per treatment (one from each batch) and 18 samples altogether. Shotgun metagenome libraries were prepared using the Celero DNA-Seq library preparation kit (NuGen, Takara Bio, USA) with enzymatic shearing, according to the manufacturer’s instructions. All libraries were then pooled in equal volumes and size selection (350–400 bp fragments) was performed using a Blue PippinPrep instrument (Sage Scientific). The libraries were then sequenced using an Illumina MiniSeq instrument employing a mid-output kit. Based on the number of reads per sample, the samples were repooled with varying volumes, and size selection was performed again using the same size range. The final size-selected pool was sequenced on an Illumina NovaSeq instrument with an S4 flow cell, employing 2 × 150 base reads. Library preparation and pooling were performed at the University of Illinois at Chicago Sequencing Core (UICSQC), and sequencing was performed by Novogene Corporation (Chula Vista, CA, USA).

In total, we obtained 310 Gb of information, with 30–44 million sequences per root sample. These sequence data were submitted to the Sequence Read Archive (SRA) of the NCBI databases under accession numbers SUB6631533 and SUB8385777, BioProject: PRJNA592741.

All reads were subjected to quality control using FastQC v0.11.3 [30] and barcode trimming using Trimmomatics v0.32 [31]. Reads were mapped to the whole wheat metagenome using Bowtie2 v2.3.5.1 [32], and mapped reads were filtered out from each sample. Then, short Illumina reads from triplicates of each nitrate treatment (30, 70 and 100 ppm) were assembled using SPADES v3.13.0 [33] into longer contigs, to create three wheat root microbiome catalogs for each treatment separately. The 30 ppm nitrate catalog had 677,271 contigs with N50 of 964 bp, 70 ppm nitrate catalog had 644,394 contigs with N50 of 971 bp, and the 100 ppm catalog had 677,271 contigs with N50 of 964 bp. Those three catalogs were combined and Prodigal v2.6.2 [34] was used for protein-coding gene prediction. To create a non-redundant set of genes, we used CD-HIT-EST software v4.8.1 [35] with a similarity threshold of 95%. Those genes were used as the root gene catalog, which included 35 million partial genes. This gene catalog was searched against the non-redundant NCBI protein database using DIAMOND sensitive algorithm v0.9.24.125 [36] to assign taxonomic and functional annotations. Results were then uploaded to MEGAN Ultimate edition software v6.15.2 [37]. The LCA (lowest common ancestor) algorithm was applied (parameters used with minimum bit-score of 70, minimum support of 5% and 30% top threshold) to compute the assignment of genes to specific taxa. For functional annotation, the Kyoto Encyclopedia of Genes and Genomes (KEGG) database [38] was used. Following annotation, to generate taxonomic and functional count tables, each library was mapped to the gene catalog with Trinity mapping software v2.8.4 [39], with Bowtie2-modified parameters (--no-unal --gbar 99999999 -k 250 --dpad 0 --mp 1,1 --np 1 --score-min L,0,−0.9 -L 20 -i S,1,0.50).

### Data analyses

Significance of interactions between CO_2_ and nitrate levels on soil and plant parameters was calculated using two-way ANOVA the least-squares method, in JMP 14 Pro software (SAS Institute Inc., Cary, NC, USA). Differences between soil and plant parameters as influenced by interactions between CO_2_ and nitrate levels was calculated using Student’s *t* test in JMP 14 Pro software and statistical significance was set at *P* < 0.05 The abundance of genes measured using qPCR, was calculated using Student’s *t* test and statistical significance was set at *P* < 0.05. All sequencing data analyses were performed using R statistical software. An nMDS (non-metric multidimensional scaling) ordination plot was constructed using R package VEGAN v.2.5-5 [40]. The data matrix was transformed using normalized count transformation, and the batch effect of three experimental cycles was removed using the DESeq2 v.1.22.2 package [41] limma::removeBatchEffect function. For community structure, ordination was generated using the Bray–Curtis dissimilarity matrix, and for community function of KEGG orthologous (KO) groups, ordination was generated using the Euclidean dissimilarity matrix. Permutational multivariate analysis was used to compute the variance between bacterial community structure or function and experimental parameters (CO_2_, nitrate and cycle), using the Adonis function in the Vegan R package [42]. For comparison of taxonomic changes and functional traits, differential abundance was estimated using DESeq2 [41] and was considered significant when the difference in abundance between genes had a FDR-adjusted *P* value < 0.05. For comparison of changes in taxonomic and functional traits, the bacterial read counts table was binned into KO groups, based on DIAMOND-MEGAN annotation. The links between gene functions (denitrification and type VI secretion system (T6SS)) and wheat root microbial community structure at the order level, represented as networks, were constructed using Cytoscape v.3.7.2 software [43].

## Results

Wheat was grown for 6 weeks in a greenhouse under 400 ppm CO_2_ (aCO_2_) or 850 ppm CO_2_ (eCO_2_), and three nitrate levels representing three possible wheat-growth-sustaining fertilization scenarios: limited (30 ppm), moderate (70 ppm) and excess (100 ppm). Soil, shoots and roots were sampled at the end of week 6. This single time point was chosen for two reasons. First, in a previous study, the difference in plant nitrogen and soil soluble nitrate contents, as influenced by CO_2_ level, was evident at the end of week 6 [25], suggesting a measurable effect on the root-surface-associated microbiome. Moreover, threefold accumulation of root-surface-associated bacteria was observed at this time point compared to all previous weeks [25]. Secondly, this time point is just before spike formation, which is known to alter root exudates, and thus may alter the root-associated microbial community [44]. The impact of spike formation on plant parameters and subsequent effects on the microbiome may therefore interfere with measuring the effects of CO_2_ and nitrate on the plant microbiome.

### Influence of eCO_2_ and nitrate levels on soil and wheat parameters

**Fig. 1:**
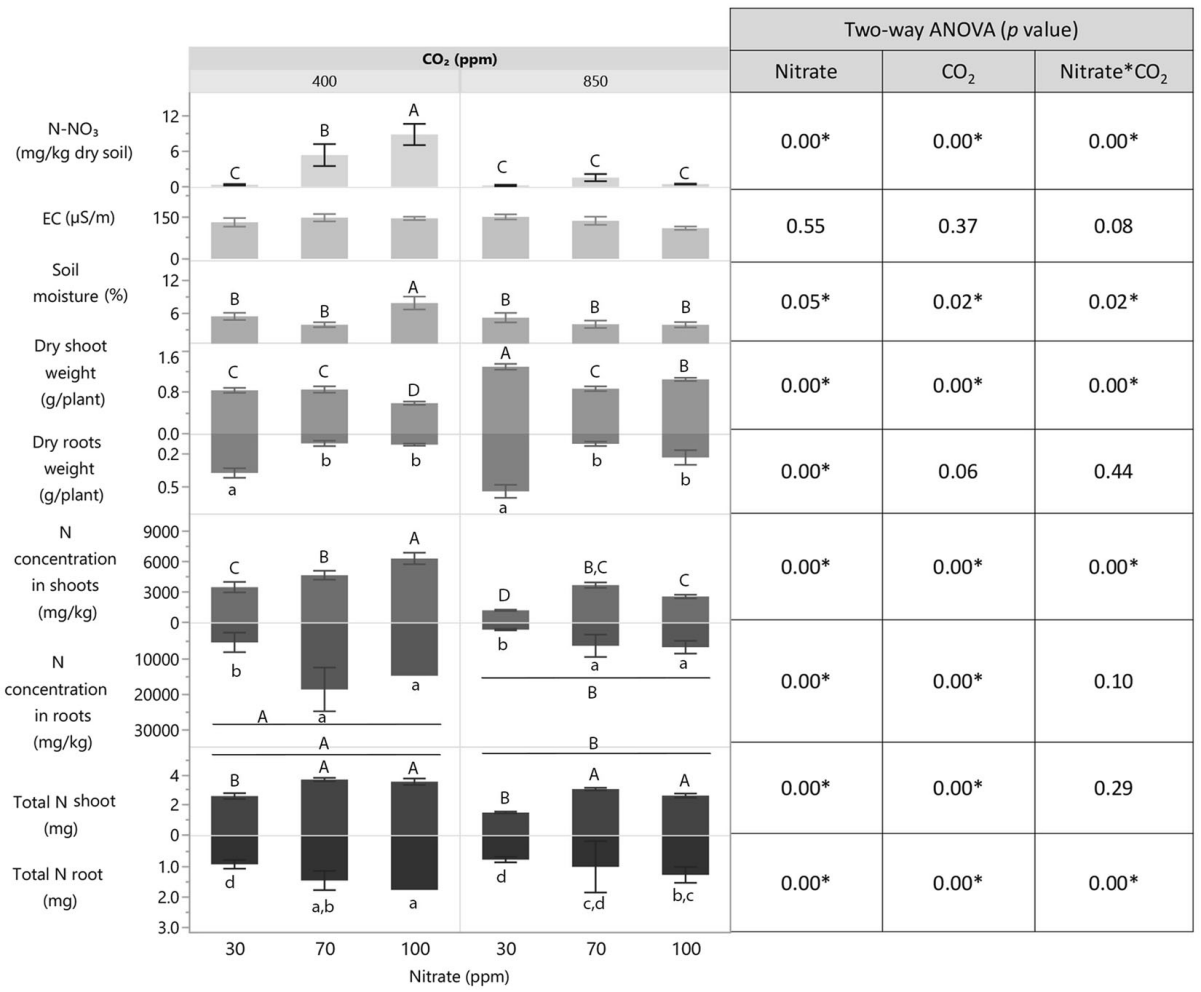
Soil and plant properties as influenced by nitrate and CO_2_ levels in 6-weeks-old wheat. Each bar represents the average of replicates with standard error (exact number of replicates can be found in Table S6). Different uppercase and lowercase letters indicate significant difference (*P* ≤ 0.05) by Student’s *t* test. Two-way ANOVA *p* values are provided in table on the right. NO-N_3_ nitrate nitrogen.

The changes in soil parameters (soluble nitrate content, electrical conductivity [EC] and moisture) and plant properties (shoot and root dry weight, nitrogen concentration and content) effected by nitrate and CO_2_ levels at the end of the 6-week period are presented in Fig. 1. Two-way ANOVA of interactions between CO_2_ and nitrate levels on soil and plant parameters was calculated. Interestingly, most of the soil and plant parameters were influenced by interactions between CO_2_ and nitrate levels (marked with an asterisk in Fig. 1). As expected, under aCO_2_, soil nitrate content was positively correlated with nitrate supply. However, under eCO_2_, soil nitrate content was negligible at all nitrate concentrations and was comparable to that under aCO_2_ and limited nitrate supply (30 ppm). Under eCO_2_, dry shoot weight increased under limited and excess nitrate, supporting the notion of plant biomass accumulation with increasing CO_2_ level. Moreover, under eCO_2_ vs. aCO_2_, plant nitrogen concentration (shoots and roots combined) and shoot total nitrogen content decreased. This indicated decreased plant nitrate accumulation with increasing CO_2_ level.

### Influence of eCO_2_ and nitrate levels on the abundance of root-surface-associated bacteria

**Fig. 2:**
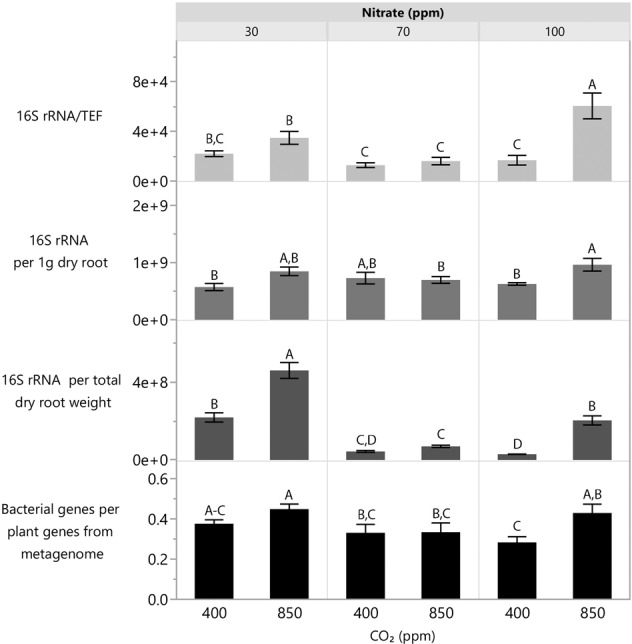
Effect of CO_2_ and nitrate levels on abundance of total bacteria per plant in roots of 6-weeks-old wheat. Gray bars represent measurement using qPCR, where each bar represents the average of nine replicates (three biological replicates, each composed of three technical ones) with standard error. Black bars represent measurement using shotgun sequencing, where each bar represents the average of three replicates (each replicate contains a mix of three technical replicates) with standard error. Different uppercase letters indicate significant difference (*P* ≤ 0.05) by Student’s *t* test.

The effect of CO_2_ and nitrate levels on the abundance of total bacteria on the roots of 6-week-old wheat plants was measured in two ways: (1) quantitative PCR (qPCR) and (2) shotgun sequencing. For the qPCR measurement, the abundance of total bacteria on the plant roots was estimated as copy numbers of the 16S rRNA gene, and calculated in three ways: (i) per plant housekeeping gene *TEF*, (ii) per 1 g dry root, and (iii) per plant, i.e., per whole root dry weight (Fig. 2). For the shotgun sequencing, the relative abundance of total bacteria per plant was calculated by dividing the total number of bacterial genes by the total number of wheat genes. All four calculations showed a similar trend: with excess (100 ppm) nitrate supply, but not 30 or 70 ppm, there was a significant increase in the relative abundance of total bacteria per plant with increasing CO_2_ level (Fig. 2). This increased proliferation of root-surface-associated bacteria may result from improved plant status, and increased availability of root exudates and a change in their composition with rising CO_2_ level, combined with the excess nitrate supply.

### Influence of eCO_2_ and nitrate levels on structure of root-surface-associated bacterial community

**Fig. 3:**
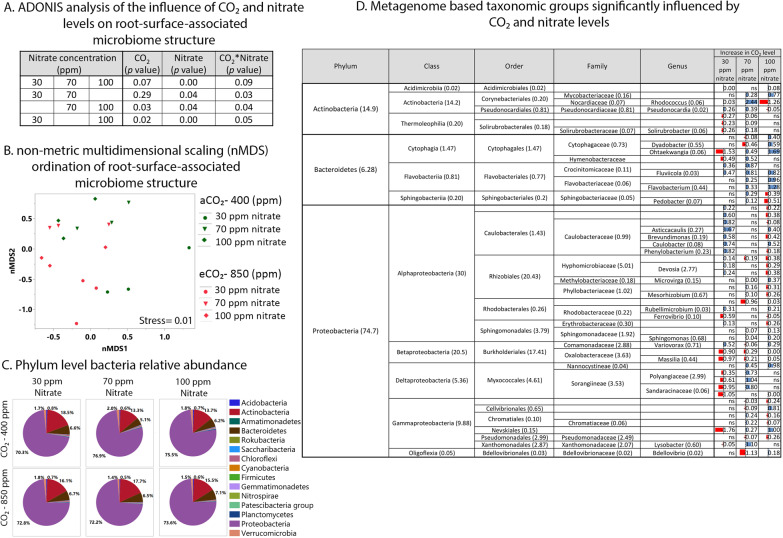
Combined influence of CO_2_ and nitrate levels on 6-weeks-old wheat root-surface-associated bacterial community structure. **a** ADONIS analysis of the effect of CO_2_ and different combinations of nitrate on bacterial community structure. **b** nMDS ordination plot showing clustering patterns of root-surface-associated bacterial community structure as influenced by all nitrate and CO_2_ levels. Data matrix was transformed using normalized count transformation and batch effect was removed using DESeq2 package, and then ordination was generated using Bray–Curtis dissimilarity. **c** Bacterial community structure at the phylum level as influenced by nitrate and CO_2_ level. Changes in microbiome gene abundance were calculated using DESeq2 with cutoff <0.05 of FDR-adjusted *P* value. **d** Significantly changed groups with increase in CO_2_ level at three nitrate levels at all taxonomic levels. Numbers in parentheses indicate relative abundance of this specific taxa out of whole phylogeny assigned from metagenome. Blue indicates positive and red indicates negative log2 fold change. *n* = 3.

After filtering out the plant host genes from those of the root-surface-associated microbiome, around 84% of the genes were related to *Bacteria*, 0.56% to *Eukaryota*, 0.06% to *Archaea*, and 14% could not be assigned to any known taxonomic group. The *Eukaryota*, *Archaea*, and unassigned genes were filtered out from the root-surface-associated microbiome and the bacterial genes were further analyzed. Bacterial community structure on the roots of 6-week-old wheat plants was significantly influenced by nitrate level (calculated using the Adonis function in the Vegan R package; *P* < 0.006), and less so by CO_2_ level (Adonis; *P* < 0.07) (Fig. 3a, b). A list of all taxonomic groups whose abundance significantly changed as a function of CO_2_ and nitrate levels is provided in Table S2. The most dominant phylum in all samples was *Proteobacteria* (70–77% relative abundance), followed by *Actinobacteria* (13–18%) and *Bacteroidetes* (5–7%) (Fig. 3c). Pairwise comparisons between supplied nitrate concentrations showed that bacterial community structure was influenced by the interaction between CO_2_ and nitrate levels (Fig. 3a). The increase in abundance of almost all taxonomic groups originated from the three most abundant phyla (*Proteobacteria*, *Actinobacteria* and *Bacteroidetes*) was depended on the CO_2_–nitrate combination (Fig. 3d). Abundance of *Actinobacteria* and *Bacteroidetes* taxonomic groups was most significantly influenced by CO_2_ level at 70 ppm nitrate, whereas abundance of *Proteobacteria* taxonomic groups was most significantly influenced by CO_2_ level at 100 ppm nitrate.

### Influence of eCO_2_ and nitrate levels on functions of root-surface-associated bacterial community

**Fig. 4:**
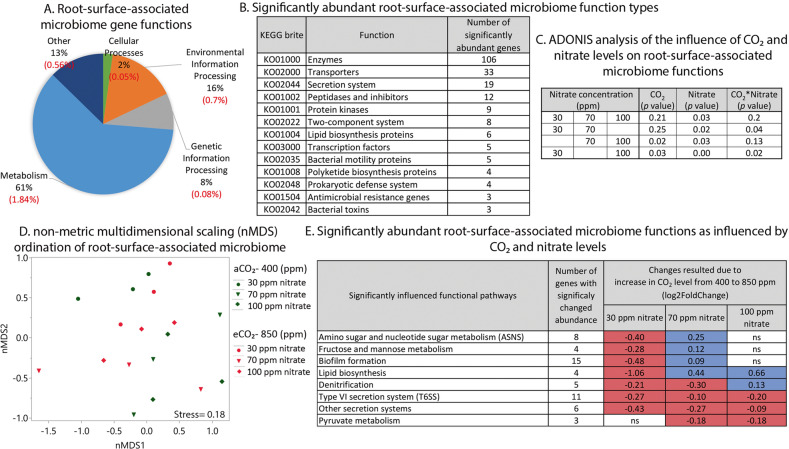
Changes in root microbiome functions (KEGG) of 6-weeks-old wheat as influenced by nitrate and CO_2_ levels. Changes in microbiome gene abundance were calculated using DESeq2 with cutoff < 0.2 of FDR-adjusted *P* value. **a** Pie chart of all functional genes in wheat root microbiome. Significantly changed pathways are marked in red. In parentheses is the percentage of that pathway out of whole metagenome which was significantly changed as a function of nitrate and CO_2_ levels. **b** Significantly abundant root-surface-associated microbiome function types. **c** ADONIS analysis of summarized effect of CO_2_ and different combinations of nitrate supply on bacterial community functional genes. **d** nMDS ordination plot showing clustering patterns of root-surface-associated microbiome functional genes as influenced by nitrate and CO_2_ levels. Data matrix was transformed using normalized count transformation and batch effect removal with the DESeq2 package, and then ordination was generated using Euclidean dissimilarity, *n* = 3. **e** Significantly abundant root-surface-associated microbiome functional gene pathways with increase in CO_2_ level. Blue indicates positive and red indicates negative log2 fold change in gene abundance between 850 and 400 ppm CO_2_. ns not significant.

**Fig. 5:**
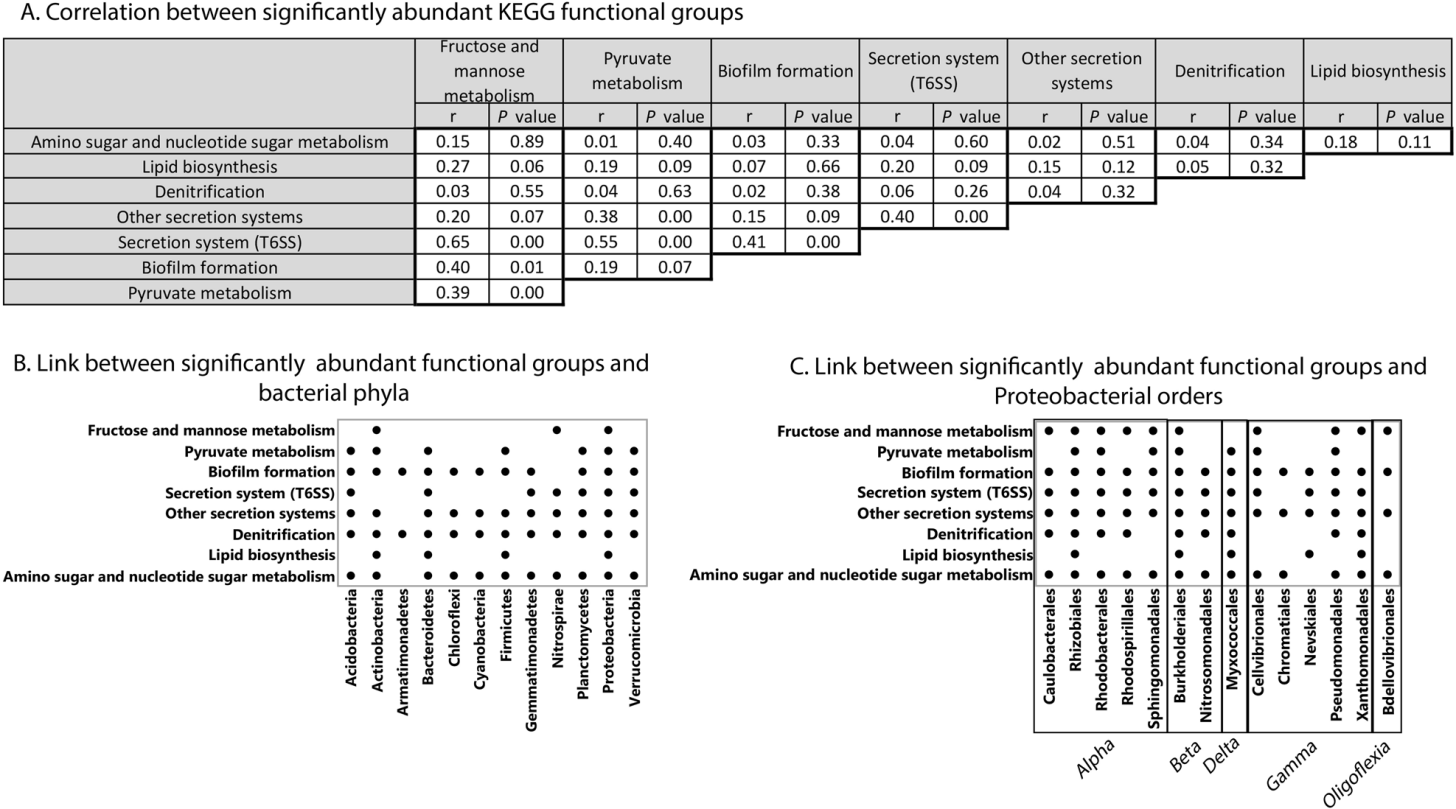
Correlation between significantly abundant root-surface-associated bacterial functional groups and association to their respective taxonomy. **a** Correlation between significantly abundant root-surface-associated bacterial functional groups. Pearson correlation (r) was calculated using Mantel test and *P* is the significance level. Link between significantly abundant root-surface-associated bacterial functional groups and bacterial phyla (**b**) and proteobacterial order level (**c**).

To reveal changes in root microbiome functions under different CO_2_ and nitrate levels, changes in microbiome gene abundance were calculated using DESeq2 with cutoff <0.2 for FDR-adjusted *P* value. More than half of the functional genes in the wheat root community metagenome were found to be involved in metabolic processes, 16% in environmental information processing, and 8% in genetic information processing (Fig. 4a). Around 3% of the functional genes of the root-surface-associated bacterial community were influenced by CO_2_ and nitrate levels. Metabolism-related KOs were the most strongly influenced group, with 1.8% out of the total 3% influenced genes. Less than 1% of the influenced genes was involved in environmental information processing and around 0.5% in other unknown processes. Half of the functional genes influenced by CO_2_ and nitrate levels encoded enzymes, 16% encoded transporters and around 10% were involved in the secretion system (Fig. 4b). A list of gene functions, divided into known pathways, whose abundance significantly changed with CO_2_ and nitrate levels, is given in Table S3. Functional genes were significantly influenced by the three nitrate levels (Adonis; *P* < 0.035) and not by CO_2_ level (Adonis; *P* < 0.21) (Fig. 4c, d). Pairwise comparisons between different combinations of supplied nitrate levels revealed that root-surface-associated bacterial community gene functions, similar to their community structures, were influenced by the interaction between CO_2_ and nitrate levels (Fig. 4c). Eight distinct functional gene groups were significantly influenced by the CO_2_–nitrate interaction (Fig. 4e): genes related to denitrification, three groups related to carbon metabolism (amino sugar and nucleotide sugar [ASNS], fructose and mannose, and pyruvate), groups related to biofilm formation, secretion systems, and in particular the type VI secretion system (T6SS), and lipid biosynthesis-related genes. Under eCO_2_ with limited (30 ppm) nitrate, the abundance of genes in seven groups (ASNS metabolism, fructose and mannose metabolism, biofilm formation, secretion systems, T6SS, lipid biosynthesis and denitrification) decreased (Fig. 4e). However, at 70 ppm nitrate, gene abundance in half of the groups increased with CO_2_ level (ASNS metabolism, fructose and mannose metabolism, biofilm formation and lipid biosynthesis) and that in the other half decreased (denitrification, pyruvate metabolism, T6SS, and other secretion systems). In the presence of excess (100 ppm) nitrate, gene abundance increased with CO_2_ level in two groups (denitrification and lipid biosynthesis), decreased in three groups (pyruvate metabolism, T6SS, and other secretion systems) and did not change in the other groups. Mantel test showed that five out of the eight significantly abundant root-surface-associated bacterial functional groups were correlated with one another under both CO_2_ levels and the three nitrate concentrations (Fig. 5a): fructose and mannose metabolism, pyruvate metabolism, biofilm formation, T6SS, and other secretion systems.

### Link between taxonomy and significantly changed functions

**Fig. 6:**
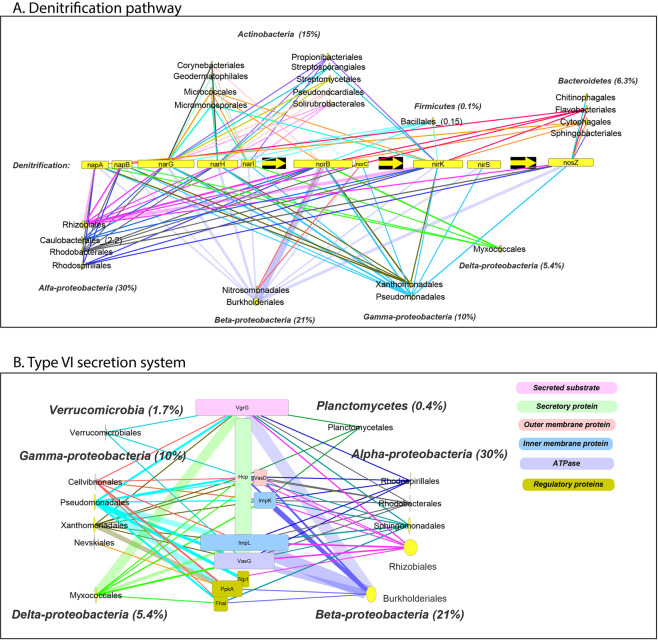
The link between selected root microbiome functions and their associated taxonomy. Link between order level root microbiome and genes of denitrification (**a**) and type VI secretion system (T6SS) (**b**). Relative abundance of each taxon under aCO_2_ and 100 ppm nitrate is represented by the size of the yellow node, and relative abundance is indicated in brackets near each taxon. Line width represents amount of gene. Width of gene rectangle indicates relative abundance of this gene in metagenome.

Bacterial taxa responsible for the eight functional groups described above are presented in Fig. 5b, c. *Proteobacteria* (as well as its two orders *Rhizobiales* and *Burkholderiales*) was the only phylum in which genes from all eight functional groups changed in abundance. To further study the link between taxonomy and these functional groups, two were selected (denitrification and T6SS) and all of their functional genes (significantly changed and others) were associated to their respective taxonomy (Fig. 6a, b). Genes involved in denitrification originated from seven distinct bacterial groups, belonging mostly to the *Actinobacteria*, *Bacteroidetes* and *Proteobacteria* (especially *Alphaproteobacteria*) (Fig. 6a). The only bacterial groups that had all functional denitrifying genes were *Burkholderiales* and *Pseudomonadales*, while *Rhizobiales* lacked *nirS*. When calculating the absolute amount of all genes necessary for denitrification per relative abundance of specific orders, *Burkholderiales* and *Pseudomonadales* had comparable numbers of genes (45 and 51, respectively), whereas *Rhizobiales* (the most abundant order) had only 12 genes (Table S4). In addition, a distinct taxonomic separation was observed for each denitrifying gene. For example, *Actinobacteria* had only nitrate reductase genes related to *nar* but not *nap*. Another gene, *nosZ*, originated predominantly from *Burkholderiales* and partly from *Pseudomonadales*, *Rhizobiales* and *Rhodobacterales*. To calculate the absolute amount of genes in a specific group, gene copy number (i.e., number of reads from a specific gene per sample normalized with DESeq2 to total number of reads per treatment) was divided by the relative abundance of the orders. *Rhodobacterales* had an absolute amount of 31 *nosZ* genes compared to 6, 5 and 0.3 in *Burkholderiales*, *Pseudomonadales* and *Rhizobiales*, respectively (Table S4).

Genes involved in T6SS originated from six distinct bacterial orders, with most reads per gene belonging to the *Proteobacteria* (*alpha, beta, gamma* and *delta*) and negligible amounts in the phyla *Verrucomicrobia* and *Planctomycetes* (Fig. 6b). The *Proteobacteria* with the most T6SS reads were *Rhizobiales*, followed by *Burkholderiales, Myxococcales* and *Pseudomonadales*; their number of T6SS reads was consistent with their relative abundance in the roots. Looking at the absolute amount of all genes required for the construction of T6SS per relative abundance of each group, *Pseudomonadales* had the highest number of genes (121), followed by *Myxococcales* (58), *Burkholderiales* (34) and *Rhizobiales* (1.2) (Table S5). Interestingly, the groups with higher relative abundance possessed lower absolute amounts of T6SS genes. Moreover, *Pseudomonadales*, with very small relative abundance of 3%, was the only order with all of the genes necessary to construct a complete T6SS.

## Discussion

Agricultural crops require nitrogen for their growth and development, especially at elevated CO_2_ levels, when plant growth is enhanced [35]. In this study, three scenarios representing nitrate fertilization regimes were compared under ambient and elevated CO_2_ levels: limited (30 ppm), moderate (70 ppm) and excess (100 ppm) nitrate.

The interaction between CO_2_ and nitrate levels significantly influenced soil parameters and plant development, as well as root-surface-associated bacterial abundance and community structure, and the abundance of bacterial genes with different functions. Limited nitrate supply resulted in negligible nitrogen concentrations in the soil at both CO_2_ levels. Under eCO_2_, nitrogen concentration and total content in the plants decreased, while shoot biomass increased (Fig. 1). Moderate nitrate supply under eCO_2_ resulted in a decrease in soil nitrate and plant total nitrogen, with no difference in biomass. Excess nitrate under eCO_2_ resulted in decreased soil nitrate and plant nitrogen with increased shoot biomass. The decrease in plant nitrogen could be because 90% of the nitrogen was supplied as nitrate and only 10% as ammonium, and it has been suggested that eCO_2_ inhibits nitrate-N assimilation into proteins in wheat [11–13]. Thus, with nitrogen mainly supplied as nitrate, its concentration generally decreases in plants under eCO_2_ [9, 10]. Indeed, in our study, under eCO_2_, nitrate fertilization did not result in nitrogen accumulation in the plants. Nevertheless, its content in the soil decreased, indicating an alternative nitrate-consumption process—probably denitrification. Indeed, the abundance of denitrification-associated functional genes in the metagenome of the root-surface-associated bacterial community increased significantly under eCO_2_ and excess nitrate (Fig. 4e).

Previous studies have shown that under eCO_2_, increased carbon availability in the soil originated from plants, lead to bacterial proliferation and activity [45, 46]. In this study, we used several methods to directly quantify the abundance of root-surface-associated bacteria. The relative abundance of total bacteria per plant increased under eCO_2_ with excess nitrate, but not with limited or moderate nitrate (Fig. 2). In addition, eCO_2_ significantly influenced the abundance of genes encoding enzymes (with changes in 3% of all enzymes in the metagenome), transporters (changes in 4% of total transporters) and the secretion system (changes in 8% of all secretion system genes). These alterations in bacterial abundance and in some of their functional genes may be the result of increased root exudates under eCO_2_, enabling bacteria to respond to the environmental shifts and proliferate.

In agreement with previous reports [22, 24, 47], the wheat root microbial community consisted mostly of *Proteobacteria*, *Actinobacteria* and *Bacteroidetes* (Fig. 3c). *Proteobacteria*, the largest taxonomic group in the wheat root-surface-associated bacterial metagenome (~ 75%), was also the group that was most influenced by eCO_2_ at all three nitrate levels (Fig. 3d). Moreover, *Proteobacteria* is the only phylum that contained genes from all of the functional gene groups affected by the CO_2_–nitrate interaction (Fig. 5a). At the order level, *Rhizobiales* was the dominant group, followed by *Burkholderiales*, *Myxococcales*, *Sphingomonadales* and *Pseudomonadales*. The relative abundance of those groups is in agreement with a recent study in which several wheat genotypes were grown under field conditions in different soil types [22]: not only was *Rhizobiales* the dominant group, but its functions seemed to play an important role in the wheat root microbiome. In another study, removing *Rhizobiales-*related functions from wheat roots in silico had the highest impact on the functional network of a root microbial community [48], whereas removing *Burkholderiales* from the network had a smaller impact, and removing *Pseudomonadales* had almost no impact at all [48]. In the current study, *Rhizobiales* and *Burkholderiales* were the only two orders of *Proteobacteria* to have all of the significantly changed functional genes, while *Pseudomonadales* lacked the significantly changed lipid biosynthesis genes (Fig. 5b). Moreover, despite the dominance of *Rhizobiales* compared to *Burkholderiales* and *Pseudomonadales*, they had less (in terms of gene number per relative abundance of the group) of the genes required for both denitrification and construction of T6SS (Tables S4 and S5). This suggests that *Burkholderiales* and *Pseudomonadales* play a major role in the functional variations occurring in the root microbiome as a result of eCO_2_ and nitrate level (the eight functional groups affected by these parameters’ interaction). While denitrification in the root microbial community is well understood, the function and role of T6SS in providing fitness and colonization advantages to root-surface-associated bacteria have only recently been investigated [49–51]. In the current study, a strong correlation was observed between five functional gene groups whose abundance was significantly changed (T6SS and other secretion systems, biofilm formation, pyruvate metabolism, and fructose and mannose metabolism) (Fig. 5a). A recent study identified acetic, succinic and malic acids as organic acids secreted by wheat roots, with succinic acid being the major organic acid exudate and its secreted concentration changing as a function of nitrogen supply [22]. Those three organic acids are linked to pyruvate metabolism (https://www.genome.jp/kegg/kegg2.html). This may support the notion that variations in root exudates resulting from eCO_2_ and nitrate levels affect colonization patterns and community assembly via interactions among root-surface-associated bacteria.

In this study, we demonstrate that interactions between CO_2_ and nitrate levels affect plant development, as well as root-surface-associated bacterial community structure and functions that probably serve for root colonization and fitness. Analyzing the connections between phylogeny and gene functions revealed that *Rhizobiales, Burkholderiales* and *Pseudomonadales* are responsible for most of the significant functional changes in the root microbiome. To the best of our knowledge, this is the first metagenomics study to shed light on alterations in soil and wheat properties and root-surface-associated bacteria as a result of combined atmospheric CO_2_ levels and nitrate fertilization. Further understanding the response of the root microbiome to changing environmental conditions may help sustain future wheat cultivation by improving fertilization to compensate for nitrate loss by denitrification. Furthermore, we believe that such studies should be carried out on other agriculturally important crops to improve yield and support growing populations, even under eCO_2_.

## Supplementary information

Supplemetary material
