# Supplementary material for: Elevated CO2 and nitrate levels increase wheat root-associated bacterial abundance and impact rhizosphere microbial community composition and function
Source: ISME J. 2020 Nov 18;15(4):1073–84. doi: 10.1038/s41396-020-00831-8 (PMC8115143; doi:10.1038/s41396-020-00831-8)
Supplement: Supplementary file 1 — Supplemetary material [file 41396_2020_831_MOESM1_ESM.docx]

| Primer name | Primer sequence (5'→3') | Amplicon  size (bp) | Master mix used for qPCR | Thermal profile of qPCR | Reference |
| --- | --- | --- | --- | --- | --- |
| 16S-331F | TCC TAC GGG AGG CAG CAG T | 195 | FAST SYBR MasterMix | 95°C for 5 min, 40 cycles at 95°C for 5 s, 60°C for 30 s,  95°C for 10 s (+0.3) | [1, 2] |
| 16S-518R | ATT ACC GCG GCT GCTG G |  |  |  |  |
| TEF_F | ACT GTG CAG TAG TAC TTG GTG | 155 | FAST SYBR MasterMix | 95°C for 5 min, 40 cycles at 95°C for 5 s, 60°C for 30 s,  95°C for 10 s (+0.3) | [3] |
| TEF_R | AAG CTA GGA GGT ATT GAC AAG |  |  |  |  |

**Table S1** Primers used for quantitative RT-PCR

1. Hunter N, Nadkarni MA, Jacques NA, Martin FE. Determination of bacterial load by real-time PCR using a broad-range (universal) probe and primers set. *Microbiology* 2002; **148**: 257–266.

2. Lopez I, Ruiz-Larrea F, Cocolin L, Orr E, Phister T, Marshall M, et al. Design and evaluation of PCR primers for analysis of bacterial populations in wine by denaturing gradient gel electrophoresis. *Appl Environ Microbiol* 2003; **69**: 6801–6807.

3. Ruppel S, Rühlmann J, Merbach W. Quantification and localization of bacteria in plant tissues using quantitative real-time PCR and online emission fingerprinting. *Plant Soil* 2006; **286**: 21–35.

**Table S2** List of all taxonomic groups of which abundance significantly changed as a function of CO_2_ and nitrate levels.

**Table S3** List of gene functions, divided into known pathways, of which abundance significantly changed as function of CO_2_ and nitrate levels.

**Table S4** Bacteria possessing functional denitrification genes

| **KEGG number** | **Gene** | **Phylum** | **Class** | **Order** | **Family** | **Genus** |
| --- | --- | --- | --- | --- | --- | --- |
| K02567 | napA | Bacteroidetes | Cytophagia | Cytophagales |  |  |
| K02567 | napA | Planctomycetes |  |  |  |  |
| K02567 | napA | Proteobacteria | Alphaproteobacteria | Rhodobacterales | Rhodobacteraceae |  |
| K02567 | napA | Proteobacteria | Alphaproteobacteria | Rhizobiales | Phyllobacteriaceae |  |
| K02567 | napA | Proteobacteria | Alphaproteobacteria | Rhizobiales | Methylobacteriaceae |  |
| K02567 | napA | Proteobacteria | Alphaproteobacteria | Rhizobiales | Rhizobiaceae | Sinorhizobium/Ensifer group |
| K02567 | napA | Proteobacteria | Alphaproteobacteria | Rhizobiales | Rhizobiaceae | Rhizobium/Agrobacterium group |
| K02567 | napA | Proteobacteria | Alphaproteobacteria | Rhizobiales | Rhizobiaceae | Sinorhizobium |
| K02567 | napA | Proteobacteria | Alphaproteobacteria | Rhodospirillales | Rhodospirillaceae |  |
| K02567 | napA | Proteobacteria | Alphaproteobacteria | Rhizobiales | Bradyrhizobiaceae | Bradyrhizobium |
| K02567 | napA | Proteobacteria | Alphaproteobacteria | Rhizobiales | Bradyrhizobiaceae |  |
| K02567 | napA | Proteobacteria | Alphaproteobacteria | Rhizobiales | Rhizobiaceae | Sinorhizobium |
| K02567 | napA | Proteobacteria | Betaproteobacteria | Burkholderiales | Burkholderiaceae |  |
| K02567 | napA | Proteobacteria | Betaproteobacteria | Burkholderiales | Alcaligenaceae |  |
| K02567 | napA | Proteobacteria | Betaproteobacteria | Burkholderiales | Comamonadaceae |  |
| K02567 | napA | Proteobacteria | Deltaproteobacteria | Myxococcales | Cystobacterineae | Archangiaceae |
| K02567 | napA | Proteobacteria | Gammaproteobacteria | Pseudomonadales | Pseudomonadaceae | Pseudomonas |
| K02567 | napA | Proteobacteria | Gammaproteobacteria | Xanthomonadales | Xanthomonadaceae |  |
| K02568 | napB | Bacteroidetes | Flavobacteriia | Flavobacteriales | Flavobacteriaceae |  |
| K02568 | napB | Proteobacteria | Alphaproteobacteria | Rhizobiales | Hyphomicrobiaceae | Devosia |
| K02568 | napB | Proteobacteria | Alphaproteobacteria | Rhizobiales | Rhizobiaceae | Rhizobium/Agrobacterium group |
| K02568 | napB | Proteobacteria | Alphaproteobacteria | Rhizobiales | Rhizobiaceae |  |
| K02568 | napB | Proteobacteria | Alphaproteobacteria | Rhizobiales | Rhizobiaceae | Sinorhizobium/Ensifer group |
| K02568 | napB | Proteobacteria | Alphaproteobacteria | Rhodospirillales | Rhodospirillaceae |  |
| K02568 | napB | Proteobacteria | Alphaproteobacteria | Rhodobacterales |  |  |
| K02568 | napB | Proteobacteria | Alphaproteobacteria | Rhizobiales | Bradyrhizobiaceae |  |
| K02568 | napB | Proteobacteria | Alphaproteobacteria | Rhizobiales | Rhizobiaceae | Sinorhizobium |
| K02568 | napB | Proteobacteria | Alphaproteobacteria | Rhizobiales |  |  |
| K02568 | napB | Proteobacteria | Betaproteobacteria | Burkholderiales | Oxalobacteraceae |  |
| K02568 | napB | Proteobacteria | Deltaproteobacteria | Myxococcales | Sorangiineae | Polyangiaceae |
| K02568 | napB | Proteobacteria | Gammaproteobacteria | Pseudomonadales | Pseudomonadaceae | Pseudomonas |
| K02568 | napB | Proteobacteria | Gammaproteobacteria | Xanthomonadales | Xanthomonadaceae |  |
| K02568 | napB | Proteobacteria | Gammaproteobacteria | Cellvibrionales | Cellvibrionaceae | Cellvibrio |
| K00370 | narG | Actinobacteria | Actinobacteria | Propionibacteriales |  |  |
| K00370 | narG | Actinobacteria | Actinobacteria | Corynebacteriales |  |  |
| K00370 | narG | Actinobacteria | Actinobacteria | Geodermatophilales | Geodermatophilaceae |  |
| K00370 | narG | Actinobacteria | Actinobacteria | Streptosporangiales |  |  |
| K00370 | narG | Actinobacteria | Actinobacteria | Micrococcales | Micrococcaceae |  |
| K00370 | narG | Actinobacteria | Actinobacteria | Propionibacteriales | Nocardioidaceae |  |
| K00370 | narG | Actinobacteria | Actinobacteria | Micrococcales | Promicromonosporaceae |  |
| K00370 | narG | Actinobacteria | Actinobacteria | Corynebacteriales | Mycobacteriaceae |  |
| K00370 | narG | Actinobacteria | Actinobacteria | Micromonosporales | Micromonosporaceae | Micromonospora |
| K00370 | narG | Actinobacteria | Actinobacteria | Pseudonocardiales | Pseudonocardiaceae |  |
| K00370 | narG | Actinobacteria | Actinobacteria | Micrococcales |  |  |
| K00370 | narG | Actinobacteria | Actinobacteria | Streptomycetales | Streptomycetaceae | Streptomyces |
| K00370 | narG | Actinobacteria | Actinobacteria | Propionibacteriales | Nocardioidaceae | Nocardioides |
| K00370 | narG | Actinobacteria | Actinobacteria | Micromonosporales | Micromonosporaceae | Actinoplanes |
| K00370 | narG | Actinobacteria | Actinobacteria | Micromonosporales | Micromonosporaceae |  |
| K00370 | narG | Actinobacteria | Thermoleophilia | Solirubrobacterales | Solirubrobacteraceae | Solirubrobacter |
| K00370 | narG | Firmicutes | Bacilli | Bacillales | Bacillaceae | Bacillus |
| K00370 | narG | Firmicutes | Bacilli | Bacillales | Bacillaceae |  |
| K00370 | narG | Firmicutes | Bacilli | Bacillales |  |  |
| K00370 | narG | Firmicutes | Bacilli | Bacillales | Paenibacillaceae | Paenibacillus |
| K00370 | narG | Nitrospirae |  |  |  |  |
| K00370 | narG | Proteobacteria | Alphaproteobacteria | Rhodobacterales | Rhodobacteraceae |  |
| K00370 | narG | Proteobacteria | Alphaproteobacteria | Caulobacterales |  |  |
| K00370 | narG | Proteobacteria | Alphaproteobacteria | Rhizobiales | Bradyrhizobiaceae |  |
| K00370 | narG | Proteobacteria | Betaproteobacteria | Burkholderiales | Burkholderiaceae |  |
| K00370 | narG | Proteobacteria | Betaproteobacteria | Burkholderiales | Alcaligenaceae |  |
| K00370 | narG | Proteobacteria | Betaproteobacteria | Burkholderiales | Comamonadaceae | Acidovorax |
| K00370 | narG | Proteobacteria | Betaproteobacteria | Burkholderiales | Oxalobacteraceae |  |
| K00370 | narG | Proteobacteria | Betaproteobacteria | Burkholderiales | Comamonadaceae |  |
| K00370 | narG | Proteobacteria | Gammaproteobacteria | Xanthomonadales | Xanthomonadaceae |  |
| K00370 | narG | Proteobacteria | Gammaproteobacteria | Pseudomonadales | Pseudomonadaceae | Pseudomonas |
| K00371 | narH | Actinobacteria | Actinobacteria | Micrococcales | Micrococcaceae | Arthrobacter |
| K00371 | narH | Actinobacteria | Actinobacteria | Micrococcales | Promicromonosporaceae |  |
| K00371 | narH | Actinobacteria | Actinobacteria | Propionibacteriales | Nocardioidaceae | Nocardioides |
| K00371 | narH | Actinobacteria | Actinobacteria | Streptosporangiales |  |  |
| K00371 | narH | Actinobacteria | Actinobacteria | Pseudonocardiales | Pseudonocardiaceae | Pseudonocardia |
| K00371 | narH | Actinobacteria | Actinobacteria | Geodermatophilales | Geodermatophilaceae |  |
| K00371 | narH | Actinobacteria | Actinobacteria | Corynebacteriales |  |  |
| K00371 | narH | Actinobacteria | Actinobacteria | Pseudonocardiales | Pseudonocardiaceae |  |
| K00371 | narH | Actinobacteria | Actinobacteria | Streptomycetales | Streptomycetaceae | Streptomyces |
| K00371 | narH | Actinobacteria | Actinobacteria | Micrococcales | Micrococcaceae |  |
| K00371 | narH | Actinobacteria | Actinobacteria | Micrococcales |  |  |
| K00371 | narH | Actinobacteria | Actinobacteria | Micromonosporales | Micromonosporaceae |  |
| K00371 | narH | Actinobacteria | Thermoleophilia | Solirubrobacterales |  |  |
| K00371 | narH | Firmicutes | Bacilli | Bacillales | Bacillaceae |  |
| K00371 | narH | Firmicutes | Bacilli | Bacillales |  |  |
| K00371 | narH | Firmicutes | Bacilli | Bacillales | Paenibacillaceae | Paenibacillus |
| K00371 | narH | Nitrospirae |  |  |  |  |
| K00371 | narH | Proteobacteria | Alphaproteobacteria | Caulobacterales |  |  |
| K00371 | narH | Proteobacteria | Alphaproteobacteria | Rhizobiales |  |  |
| K00371 | narH | Proteobacteria | Alphaproteobacteria | Rhodobacterales | Rhodobacteraceae |  |
| K00371 | narH | Proteobacteria | Betaproteobacteria | Burkholderiales | Alcaligenaceae |  |
| K00371 | narH | Proteobacteria | Betaproteobacteria | Burkholderiales | Oxalobacteraceae |  |
| K00371 | narH | Proteobacteria | Betaproteobacteria | Burkholderiales | Comamonadaceae |  |
| K00371 | narH | Proteobacteria | Betaproteobacteria | Burkholderiales | Comamonadaceae | Acidovorax |
| K00371 | narH | Proteobacteria | Gammaproteobacteria | Pseudomonadales |  |  |
| K00371 | narH | Proteobacteria | Gammaproteobacteria | Xanthomonadales | Xanthomonadaceae |  |
| K00371 | narH | Proteobacteria | Gammaproteobacteria | Pseudomonadales | Pseudomonadaceae | Pseudomonas |
| K00374 | narI | Actinobacteria | Actinobacteria | Corynebacteriales |  |  |
| K00374 | narI | Actinobacteria | Actinobacteria | Micrococcales | Micrococcaceae | Arthrobacter |
| K00374 | narI | Actinobacteria | Actinobacteria | Geodermatophilales | Geodermatophilaceae |  |
| K00374 | narI | Actinobacteria | Actinobacteria | Micrococcales | Promicromonosporaceae |  |
| K00374 | narI | Actinobacteria | Actinobacteria | Propionibacteriales | Nocardioidaceae | Nocardioides |
| K00374 | narI | Actinobacteria | Actinobacteria | Micrococcales |  |  |
| K00374 | narI | Actinobacteria | Actinobacteria | Micromonosporales | Micromonosporaceae | Micromonospora |
| K00374 | narI | Actinobacteria | Actinobacteria | Micromonosporales | Micromonosporaceae |  |
| K00374 | narI | Actinobacteria | Thermoleophilia | Solirubrobacterales |  |  |
| K00374 | narI | Firmicutes | Bacilli | Bacillales | Paenibacillaceae |  |
| K00374 | narI | Firmicutes | Bacilli | Bacillales | Paenibacillaceae | Paenibacillus |
| K00374 | narI | Proteobacteria | Alphaproteobacteria | Rhizobiales | Bradyrhizobiaceae |  |
| K00374 | narI | Proteobacteria | Betaproteobacteria | Burkholderiales | Alcaligenaceae |  |
| K00374 | narI | Proteobacteria | Betaproteobacteria | Burkholderiales | Oxalobacteraceae |  |
| K00374 | narI | Proteobacteria | Betaproteobacteria | Burkholderiales | Comamonadaceae |  |
| K00374 | narI | Proteobacteria | Betaproteobacteria | Burkholderiales | Comamonadaceae | Acidovorax |
| K00374 | narI | Proteobacteria | Betaproteobacteria | Burkholderiales |  |  |
| K00374 | narI | Proteobacteria | Deltaproteobacteria | Myxococcales | Cystobacterineae | Myxococcaceae |
| K00374 | narI | Proteobacteria | Gammaproteobacteria | Pseudomonadales | Pseudomonadaceae | Pseudomonas |
| K00368 | nirK | Actinobacteria | Acidimicrobiia | Acidimicrobiales |  |  |
| K00368 | nirK | Actinobacteria | Actinobacteria | Micrococcales |  |  |
| K00368 | nirK | Actinobacteria | Actinobacteria | Propionibacteriales |  |  |
| K00368 | nirK | Actinobacteria | Actinobacteria | Micrococcales | Promicromonosporaceae |  |
| K00368 | nirK | Actinobacteria | Actinobacteria | Propionibacteriales | Nocardioidaceae | Nocardioides |
| K00368 | nirK | Actinobacteria | Actinobacteria | Streptosporangiales |  |  |
| K00368 | nirK | Actinobacteria | Actinobacteria | Micromonosporales | Micromonosporaceae |  |
| K00368 | nirK | Bacteroidetes | Cytophagia | Cytophagales | Hymenobacteraceae |  |
| K00368 | nirK | Bacteroidetes | Flavobacteriia | Flavobacteriales | Flavobacteriaceae |  |
| K00368 | nirK | Bacteroidetes | Flavobacteriia | Flavobacteriales | Flavobacteriaceae | Flavobacterium |
| K00368 | nirK | Bacteroidetes | Flavobacteriia | Flavobacteriales |  |  |
| K00368 | nirK | Chloroflexi | Chloroflexia | Herpetosiphonales | Herpetosiphonaceae | Herpetosiphon |
| K00368 | nirK | Firmicutes | Bacilli | Bacillales | Bacillaceae |  |
| K00368 | nirK | Firmicutes | Bacilli | Bacillales | Bacillaceae | Bacillus |
| K00368 | nirK | Gemmatimonadetes |  |  |  |  |
| K00368 | nirK | Nitrospirae |  |  |  |  |
| K00368 | nirK | Proteobacteria | Alphaproteobacteria | Caulobacterales | Caulobacteraceae |  |
| K00368 | nirK | Proteobacteria | Alphaproteobacteria | Rhizobiales | Bradyrhizobiaceae |  |
| K00368 | nirK | Proteobacteria | Alphaproteobacteria | Caulobacterales |  |  |
| K00368 | nirK | Proteobacteria | Alphaproteobacteria | Rhizobiales | Phyllobacteriaceae |  |
| K00368 | nirK | Proteobacteria | Alphaproteobacteria | Rhodobacterales | Rhodobacteraceae |  |
| K00368 | nirK | Proteobacteria | Alphaproteobacteria | Rhodospirillales | Rhodospirillaceae |  |
| K00368 | nirK | Proteobacteria | Alphaproteobacteria | Rhodobacterales |  |  |
| K00368 | nirK | Proteobacteria | Alphaproteobacteria | Rhizobiales | Rhizobiaceae |  |
| K00368 | nirK | Proteobacteria | Alphaproteobacteria | Rhizobiales | Rhizobiaceae | Sinorhizobium/Ensifer group |
| K00368 | nirK | Proteobacteria | Alphaproteobacteria | Rhizobiales | Bradyrhizobiaceae | Bradyrhizobium |
| K00368 | nirK | Proteobacteria | Alphaproteobacteria | Rhizobiales | Rhizobiaceae | Rhizobium/Agrobacterium group |
| K00368 | nirK | Proteobacteria | Alphaproteobacteria | Rhizobiales |  |  |
| K00368 | nirK | Proteobacteria | Betaproteobacteria | Burkholderiales | Alcaligenaceae |  |
| K00368 | nirK | Proteobacteria | Betaproteobacteria | Burkholderiales |  |  |
| K00368 | nirK | Proteobacteria | Betaproteobacteria | Burkholderiales | Burkholderiaceae |  |
| K00368 | nirK | Proteobacteria | Betaproteobacteria | Burkholderiales |  |  |
| K00368 | nirK | Proteobacteria | Gammaproteobacteria | Pseudomonadales |  |  |
| K00368 | nirK | Proteobacteria | Gammaproteobacteria | Xanthomonadales | Xanthomonadaceae | Lysobacter |
| K00368 | nirK | Proteobacteria | Gammaproteobacteria | Xanthomonadales |  |  |
| K00368 | nirK | Proteobacteria | Gammaproteobacteria | Xanthomonadales | Xanthomonadaceae |  |
| K00368 | nirK | Verrucomicrobia | Opitutae | Opitutales | Opitutaceae |  |
| K15864 | nirS | Chloroflexi | Chloroflexia | Chloroflexales |  |  |
| K15864 | nirS | Proteobacteria | Alphaproteobacteria |  |  |  |
| K15864 | nirS | Proteobacteria | Betaproteobacteria | Burkholderiales |  |  |
| K15864 | nirS | Proteobacteria | Gammaproteobacteria | Pseudomonadales | Pseudomonadaceae | Pseudomonas |
| K04561 | norB | Actinobacteria | Actinobacteria | Corynebacteriales | Mycobacteriaceae |  |
| K04561 | norB | Actinobacteria | Actinobacteria | Micrococcales |  |  |
| K04561 | norB | Bacteroidetes | Cytophagia | Cytophagales | Hymenobacteraceae |  |
| K04561 | norB | Bacteroidetes | Cytophagia | Cytophagales |  |  |
| K04561 | norB | Bacteroidetes | Flavobacteriia | Flavobacteriales | Flavobacteriaceae | Flavobacterium |
| K04561 | norB | Cyanobacteria |  |  |  |  |
| K04561 | norB | Firmicutes | Bacilli | Bacillales | Bacillaceae |  |
| K04561 | norB | Firmicutes | Bacilli | Bacillales | Bacillaceae | Bacillus |
| K04561 | norB | Planctomycetes | Planctomycetia | Planctomycetales | Planctomycetaceae |  |
| K04561 | norB | Planctomycetes |  |  |  |  |
| K04561 | norB | Proteobacteria | Alphaproteobacteria | Rhodobacterales |  |  |
| K04561 | norB | Proteobacteria | Alphaproteobacteria | Rhodospirillales | Rhodospirillaceae |  |
| K04561 | norB | Proteobacteria | Alphaproteobacteria | Caulobacterales |  |  |
| K04561 | norB | Proteobacteria | Alphaproteobacteria | Rhizobiales | Bradyrhizobiaceae | Bradyrhizobium |
| K04561 | norB | Proteobacteria | Alphaproteobacteria | Rhizobiales | Rhizobiaceae | Sinorhizobium |
| K04561 | norB | Proteobacteria | Alphaproteobacteria | Rhizobiales | Bradyrhizobiaceae |  |
| K04561 | norB | Proteobacteria | Alphaproteobacteria | Rhizobiales | Rhizobiaceae | Rhizobium/Agrobacterium group |
| K04561 | norB | Proteobacteria | Alphaproteobacteria | Rhizobiales | Rhizobiaceae | Sinorhizobium/Ensifer group |
| K04561 | norB | Proteobacteria | Alphaproteobacteria | Rhizobiales | Rhizobiaceae |  |
| K04561 | norB | Proteobacteria | Alphaproteobacteria | Rhodobacterales | Rhodobacteraceae |  |
| K04561 | norB | Proteobacteria | Alphaproteobacteria | Rhizobiales |  |  |
| K04561 | norB | Proteobacteria | Betaproteobacteria | Burkholderiales | Comamonadaceae | Variovorax |
| K04561 | norB | Proteobacteria | Betaproteobacteria | Burkholderiales | unclassified Burkholderiales |  |
| K04561 | norB | Proteobacteria | Betaproteobacteria | Burkholderiales | Comamonadaceae | Acidovorax |
| K04561 | norB | Proteobacteria | Betaproteobacteria | Nitrosomonadales |  |  |
| K04561 | norB | Proteobacteria | Betaproteobacteria | Burkholderiales | Burkholderiaceae |  |
| K04561 | norB | Proteobacteria | Betaproteobacteria | Burkholderiales | Comamonadaceae |  |
| K04561 | norB | Proteobacteria | Deltaproteobacteria | Myxococcales | Cystobacterineae | Archangiaceae |
| K04561 | norB | Proteobacteria | Deltaproteobacteria | Myxococcales | Cystobacterineae |  |
| K04561 | norB | Proteobacteria | Deltaproteobacteria | Myxococcales | Sorangiineae | Polyangiaceae |
| K04561 | norB | Proteobacteria | Gammaproteobacteria | Pseudomonadales |  |  |
| K04561 | norB | Proteobacteria | Gammaproteobacteria | Xanthomonadales | Xanthomonadaceae | Pseudoxanthomonas |
| K04561 | norB | Proteobacteria | Gammaproteobacteria | Xanthomonadales |  |  |
| K04561 | norB | Proteobacteria | Gammaproteobacteria | Xanthomonadales | Xanthomonadaceae |  |
| K04561 | norB | Proteobacteria | Gammaproteobacteria | Pseudomonadales | Pseudomonadaceae | Pseudomonas |
| K02305 | norC | Bacteroidetes | Flavobacteriia | Flavobacteriales | Flavobacteriaceae | Flavobacterium |
| K02305 | norC | Firmicutes |  |  |  |  |
| K02305 | norC | Proteobacteria | Alphaproteobacteria | Rhizobiales | Rhizobiaceae |  |
| K02305 | norC | Proteobacteria | Alphaproteobacteria | Rhodobacterales | Rhodobacteraceae |  |
| K02305 | norC | Proteobacteria | Alphaproteobacteria | Rhodobacterales |  |  |
| K02305 | norC | Proteobacteria | Alphaproteobacteria | Rhizobiales | Rhizobiaceae | Sinorhizobium/Ensifer group |
| K02305 | norC | Proteobacteria | Alphaproteobacteria | Rhizobiales |  |  |
| K02305 | norC | Proteobacteria | Betaproteobacteria | Nitrosomonadales |  |  |
| K02305 | norC | Proteobacteria | Betaproteobacteria | Burkholderiales |  |  |
| K02305 | norC | Proteobacteria | Gammaproteobacteria | Pseudomonadales |  |  |
| K02305 | norC | Proteobacteria | Gammaproteobacteria | Pseudomonadales | Pseudomonadaceae | Pseudomonas |
| K00376 | nosZ | Bacteroidetes | Chitinophagia | Chitinophagales | Chitinophagaceae |  |
| K00376 | nosZ | Bacteroidetes | Cytophagia | Cytophagales |  |  |
| K00376 | nosZ | Bacteroidetes | Cytophagia | Cytophagales | Hymenobacteraceae |  |
| K00376 | nosZ | Bacteroidetes | Flavobacteriia | Flavobacteriales | Flavobacteriaceae | Flavobacterium |
| K00376 | nosZ | Bacteroidetes | Sphingobacteriia | Sphingobacteriales | Sphingobacteriaceae |  |
| K00376 | nosZ | Proteobacteria | Alphaproteobacteria | Rhodospirillales | Rhodospirillaceae |  |
| K00376 | nosZ | Proteobacteria | Alphaproteobacteria | Rhodobacterales |  |  |
| K00376 | nosZ | Proteobacteria | Alphaproteobacteria | Rhizobiales | Rhizobiaceae | Sinorhizobium |
| K00376 | nosZ | Proteobacteria | Alphaproteobacteria | Rhizobiales | Rhizobiaceae | Sinorhizobium/Ensifer group |
| K00376 | nosZ | Proteobacteria | Alphaproteobacteria | Rhizobiales | Bradyrhizobiaceae | Bradyrhizobium |
| K00376 | nosZ | Proteobacteria | Alphaproteobacteria | Rhizobiales | Rhizobiaceae |  |
| K00376 | nosZ | Proteobacteria | Alphaproteobacteria | Rhodobacterales | Rhodobacteraceae |  |
| K00376 | nosZ | Proteobacteria | Betaproteobacteria | Burkholderiales | Comamonadaceae |  |
| K00376 | nosZ | Proteobacteria | Betaproteobacteria | Burkholderiales | Alcaligenaceae |  |
| K00376 | nosZ | Proteobacteria | Gammaproteobacteria | Pseudomonadales | Pseudomonadaceae | Pseudomonas |

**Table S5** Bacteria possessing type 6 secretion system genes

| **KEGG number** | **Gene** | **Phylum** | **Class** | **Order** | **Family** | **Genus** |
| --- | --- | --- | --- | --- | --- | --- |
| K11913 | type VI secretion system protein | Proteobacteria | Alphaproteobacteria | Sphingomonadales |  |  |
| K11913 | type VI secretion system protein | Proteobacteria | Alphaproteobacteria | Sphingomonadales | Erythrobacteraceae |  |
| K11913 | type VI secretion system protein | Proteobacteria | Alphaproteobacteria | Sphingomonadales | Sphingomonadaceae |  |
| K11913 | type VI secretion system protein | Proteobacteria | Alphaproteobacteria | Sphingomonadales | Sphingomonadaceae | Sphingomonas |
| K11913 | type VI secretion system protein | Proteobacteria | Betaproteobacteria | Burkholderiales |  |  |
| K11905 | type VI secretion system protein | Proteobacteria | Deltaproteobacteria | Myxococcales |  |  |
| K11905 | type VI secretion system protein | Proteobacteria | Deltaproteobacteria | Myxococcales | Cystobacterineae |  |
| K11905 | type VI secretion system protein | Proteobacteria | Deltaproteobacteria | Myxococcales | Cystobacterineae | Archangiaceae |
| K11905 | type VI secretion system protein | Proteobacteria | Deltaproteobacteria | Myxococcales | Cystobacterineae | Myxococcaceae |
| K11905 | type VI secretion system protein | Proteobacteria | Deltaproteobacteria | Myxococcales | Nannocystineae | Nannocystaceae |
| K11905 | type VI secretion system protein | Proteobacteria | Deltaproteobacteria | Myxococcales | Sorangiineae |  |
| K11905 | type VI secretion system protein | Proteobacteria | Deltaproteobacteria | Myxococcales | Sorangiineae | Polyangiaceae |
| K11913 | type VI secretion system protein | Proteobacteria | Deltaproteobacteria | Myxococcales |  |  |
| K11913 | type VI secretion system protein | Proteobacteria | Deltaproteobacteria | Myxococcales | Cystobacterineae | Archangiaceae |
| K11905 | type VI secretion system protein | Proteobacteria | Gammaproteobacteria | Pseudomonadales | Pseudomonadaceae | Pseudomonas |
| K11913 | type VI secretion system protein | Proteobacteria | Gammaproteobacteria | Cellvibrionales | Cellvibrionaceae | Cellvibrio |
| K11913 | type VI secretion system protein | Proteobacteria | Gammaproteobacteria | Nevskiales | Sinobacteraceae | Steroidobacter |
| K11913 | type VI secretion system protein | Proteobacteria | Gammaproteobacteria | Nevskiales | Sinobacteraceae | Steroidobacter |
| K11913 | type VI secretion system protein | Proteobacteria | Gammaproteobacteria | Pseudomonadales | Pseudomonadaceae | Pseudomonas |
| K11902 | type VI secretion system protein ImpA | Gemmatimonadetes |  |  |  |  |
| K11902 | type VI secretion system protein ImpA | Proteobacteria | Alphaproteobacteria | Rhizobiales |  |  |
| K11902 | type VI secretion system protein ImpA | Proteobacteria | Alphaproteobacteria | Rhizobiales | Hyphomicrobiaceae | Devosia |
| K11902 | type VI secretion system protein ImpA | Proteobacteria | Alphaproteobacteria | Rhizobiales | Phyllobacteriaceae |  |
| K11902 | type VI secretion system protein ImpA | Proteobacteria | Alphaproteobacteria | Rhizobiales | Phyllobacteriaceae | Mesorhizobium |
| K11902 | type VI secretion system protein ImpA | Proteobacteria | Alphaproteobacteria | Rhizobiales | Rhizobiaceae | Sinorhizobium/Ensifer group |
| K11902 | type VI secretion system protein ImpA | Proteobacteria | Alphaproteobacteria | Rhodobacterales | Rhodobacteraceae |  |
| K11902 | type VI secretion system protein ImpA | Proteobacteria | Alphaproteobacteria | Rhodospirillales |  |  |
| K11902 | type VI secretion system protein ImpA | Proteobacteria | Alphaproteobacteria | Sphingomonadales | Erythrobacteraceae |  |
| K11902 | type VI secretion system protein ImpA | Proteobacteria | Betaproteobacteria | Burkholderiales |  |  |
| K11902 | type VI secretion system protein ImpA | Proteobacteria | Betaproteobacteria | Burkholderiales | Burkholderiaceae |  |
| K11902 | type VI secretion system protein ImpA | Proteobacteria | Betaproteobacteria | Burkholderiales | Comamonadaceae |  |
| K11902 | type VI secretion system protein ImpA | Proteobacteria | Betaproteobacteria | Burkholderiales | Comamonadaceae | Variovorax |
| K11902 | type VI secretion system protein ImpA | Proteobacteria | Betaproteobacteria | Burkholderiales | Oxalobacteraceae |  |
| K11902 | type VI secretion system protein ImpA | Proteobacteria | Betaproteobacteria | Burkholderiales | Oxalobacteraceae | Massilia |
| K11902 | type VI secretion system protein ImpA | Proteobacteria | Betaproteobacteria | Burkholderiales |  |  |
| K11902 | type VI secretion system protein ImpA | Proteobacteria | Deltaproteobacteria | Myxococcales | Sorangiineae |  |
| K11902 | type VI secretion system protein ImpA | Proteobacteria | Deltaproteobacteria | Myxococcales | Sorangiineae | Polyangiaceae |
| K11902 | type VI secretion system protein ImpA | Proteobacteria | Deltaproteobacteria | Myxococcales | Sorangiineae | Polyangiaceae |
| K11902 | type VI secretion system protein ImpA | Proteobacteria | Gammaproteobacteria | Cellvibrionales | Cellvibrionaceae |  |
| K11902 | type VI secretion system protein ImpA | Proteobacteria | Gammaproteobacteria | Cellvibrionales | Cellvibrionaceae | Cellvibrio |
| K11902 | type VI secretion system protein ImpA | Proteobacteria | Gammaproteobacteria | Pseudomonadales |  |  |
| K11902 | type VI secretion system protein ImpA | Proteobacteria | Gammaproteobacteria | Pseudomonadales | Pseudomonadaceae | Pseudomonas |
| K11902 | type VI secretion system protein ImpA | Proteobacteria | Gammaproteobacteria | Xanthomonadales | Xanthomonadaceae |  |
| K11902 | type VI secretion system protein ImpA | Proteobacteria | Gammaproteobacteria | Xanthomonadales | Xanthomonadaceae | Lysobacter |
| K11901 | type VI secretion system protein ImpB | Proteobacteria | Alphaproteobacteria | Rhizobiales |  |  |
| K11901 | type VI secretion system protein ImpB | Proteobacteria | Alphaproteobacteria | Rhizobiales | Bradyrhizobiaceae | Bradyrhizobium |
| K11901 | type VI secretion system protein ImpB | Proteobacteria | Alphaproteobacteria | Rhizobiales | Phyllobacteriaceae |  |
| K11901 | type VI secretion system protein ImpB | Proteobacteria | Alphaproteobacteria | Rhizobiales | Rhizobiaceae |  |
| K11901 | type VI secretion system protein ImpB | Proteobacteria | Alphaproteobacteria | Rhodobacterales | Rhodobacteraceae |  |
| K11901 | type VI secretion system protein ImpB | Proteobacteria | Alphaproteobacteria | Rhodospirillales |  |  |
| K11901 | type VI secretion system protein ImpB | Proteobacteria | Alphaproteobacteria | Sphingomonadales |  |  |
| K11901 | type VI secretion system protein ImpB | Proteobacteria | Betaproteobacteria | Burkholderiales |  |  |
| K11901 | type VI secretion system protein ImpB | Proteobacteria | Betaproteobacteria | Burkholderiales | Comamonadaceae |  |
| K11901 | type VI secretion system protein ImpB | Proteobacteria | Betaproteobacteria | Burkholderiales | Comamonadaceae | Variovorax |
| K11901 | type VI secretion system protein ImpB | Proteobacteria | Betaproteobacteria | Burkholderiales | Oxalobacteraceae |  |
| K11901 | type VI secretion system protein ImpB | Proteobacteria | Betaproteobacteria | Burkholderiales | Oxalobacteraceae | Massilia |
| K11901 | type VI secretion system protein ImpB | Proteobacteria | Betaproteobacteria | Burkholderiales |  |  |
| K11901 | type VI secretion system protein ImpB | Proteobacteria | Deltaproteobacteria | Myxococcales |  |  |
| K11901 | type VI secretion system protein ImpB | Proteobacteria | Deltaproteobacteria | Myxococcales | Cystobacterineae |  |
| K11901 | type VI secretion system protein ImpB | Proteobacteria | Deltaproteobacteria | Myxococcales | Nannocystineae | Nannocystaceae |
| K11901 | type VI secretion system protein ImpB | Proteobacteria | Deltaproteobacteria | Myxococcales | Sorangiineae |  |
| K11901 | type VI secretion system protein ImpB | Proteobacteria | Deltaproteobacteria | Myxococcales | Sorangiineae | Polyangiaceae |
| K11901 | type VI secretion system protein ImpB | Proteobacteria | Gammaproteobacteria | Pseudomonadales |  |  |
| K11901 | type VI secretion system protein ImpB | Proteobacteria | Gammaproteobacteria | Pseudomonadales | Pseudomonadaceae | Pseudomonas |
| K11901 | type VI secretion system protein ImpB | Verrucomicrobia | Verrucomicrobiae | Verrucomicrobiales | Verrucomicrobiaceae | Haloferula |
| K11900 | type VI secretion system protein ImpC | Proteobacteria | Alphaproteobacteria | Rhizobiales |  |  |
| K11900 | type VI secretion system protein ImpC | Proteobacteria | Alphaproteobacteria | Rhizobiales | Phyllobacteriaceae |  |
| K11900 | type VI secretion system protein ImpC | Proteobacteria | Alphaproteobacteria | Rhizobiales | Phyllobacteriaceae | Mesorhizobium |
| K11900 | type VI secretion system protein ImpC | Proteobacteria | Alphaproteobacteria | Rhizobiales | Rhizobiaceae |  |
| K11900 | type VI secretion system protein ImpC | Proteobacteria | Alphaproteobacteria | Rhodobacterales | Rhodobacteraceae |  |
| K11900 | type VI secretion system protein ImpC | Proteobacteria | Alphaproteobacteria | Rhodospirillales |  |  |
| K11900 | type VI secretion system protein ImpC | Proteobacteria | Alphaproteobacteria | Sphingomonadales |  |  |
| K11900 | type VI secretion system protein ImpC | Proteobacteria | Alphaproteobacteria | Sphingomonadales | Erythrobacteraceae |  |
| K11900 | type VI secretion system protein ImpC | Proteobacteria | Betaproteobacteria | Burkholderiales |  |  |
| K11900 | type VI secretion system protein ImpC | Proteobacteria | Betaproteobacteria | Burkholderiales | Burkholderiaceae |  |
| K11900 | type VI secretion system protein ImpC | Proteobacteria | Betaproteobacteria | Burkholderiales | Comamonadaceae |  |
| K11900 | type VI secretion system protein ImpC | Proteobacteria | Betaproteobacteria | Burkholderiales | Comamonadaceae | Acidovorax |
| K11900 | type VI secretion system protein ImpC | Proteobacteria | Betaproteobacteria | Burkholderiales | Comamonadaceae | Variovorax |
| K11900 | type VI secretion system protein ImpC | Proteobacteria | Betaproteobacteria | Burkholderiales | Oxalobacteraceae |  |
| K11900 | type VI secretion system protein ImpC | Proteobacteria | Betaproteobacteria | Burkholderiales | Oxalobacteraceae | Massilia |
| K11900 | type VI secretion system protein ImpC | Proteobacteria | Betaproteobacteria | Burkholderiales |  |  |
| K11900 | type VI secretion system protein ImpC | Proteobacteria | Deltaproteobacteria | Myxococcales |  |  |
| K11900 | type VI secretion system protein ImpC | Proteobacteria | Deltaproteobacteria | Myxococcales | Cystobacterineae |  |
| K11900 | type VI secretion system protein ImpC | Proteobacteria | Deltaproteobacteria | Myxococcales | Cystobacterineae | Archangiaceae |
| K11900 | type VI secretion system protein ImpC | Proteobacteria | Deltaproteobacteria | Myxococcales | Nannocystineae | Nannocystaceae |
| K11900 | type VI secretion system protein ImpC | Proteobacteria | Deltaproteobacteria | Myxococcales | Sorangiineae |  |
| K11900 | type VI secretion system protein ImpC | Proteobacteria | Deltaproteobacteria | Myxococcales | Sorangiineae | Polyangiaceae |
| K11900 | type VI secretion system protein ImpC | Proteobacteria | Deltaproteobacteria | Myxococcales | Sorangiineae | Polyangiaceae |
| K11900 | type VI secretion system protein ImpC | Proteobacteria | Deltaproteobacteria | Myxococcales |  |  |
| K11900 | type VI secretion system protein ImpC | Proteobacteria | Gammaproteobacteria | Cellvibrionales |  |  |
| K11900 | type VI secretion system protein ImpC | Proteobacteria | Gammaproteobacteria | Pseudomonadales |  |  |
| K11900 | type VI secretion system protein ImpC | Proteobacteria | Gammaproteobacteria | Pseudomonadales | Pseudomonadaceae | Pseudomonas |
| K11900 | type VI secretion system protein ImpC | Proteobacteria | Gammaproteobacteria | Xanthomonadales | Xanthomonadaceae |  |
| K11900 | type VI secretion system protein ImpC | Verrucomicrobia | Verrucomicrobiae | Verrucomicrobiales | Verrucomicrobiaceae | Haloferula |
| K11899 | type VI secretion system protein ImpD | Proteobacteria | Alphaproteobacteria | Rhizobiales |  |  |
| K11899 | type VI secretion system protein ImpD | Proteobacteria | Alphaproteobacteria | Rhizobiales | Rhizobiaceae |  |
| K11899 | type VI secretion system protein ImpD | Proteobacteria | Alphaproteobacteria | Rhizobiales | Rhizobiaceae | Rhizobium/Agrobacterium group |
| K11899 | type VI secretion system protein ImpD | Proteobacteria | Gammaproteobacteria | Cellvibrionales | Cellvibrionaceae | Cellvibrio |
| K11898 | type VI secretion system protein ImpE | Planctomycetes | Planctomycetia | Planctomycetales | Gemmataceae |  |
| K11898 | type VI secretion system protein ImpE | Proteobacteria | Alphaproteobacteria | Rhizobiales |  |  |
| K11898 | type VI secretion system protein ImpE | Proteobacteria | Alphaproteobacteria | Rhizobiales | Rhizobiaceae | Rhizobium/Agrobacterium group |
| K11898 | type VI secretion system protein ImpE | Proteobacteria | Alphaproteobacteria | Rhodospirillales |  |  |
| K11898 | type VI secretion system protein ImpE | Proteobacteria | Alphaproteobacteria | Sphingomonadales |  |  |
| K11898 | type VI secretion system protein ImpE | Proteobacteria | Alphaproteobacteria | Sphingomonadales | Erythrobacteraceae |  |
| K11898 | type VI secretion system protein ImpE | Proteobacteria | Betaproteobacteria | Burkholderiales |  |  |
| K11898 | type VI secretion system protein ImpE | Proteobacteria | Betaproteobacteria | Burkholderiales | Alcaligenaceae |  |
| K11898 | type VI secretion system protein ImpE | Proteobacteria | Betaproteobacteria | Burkholderiales | Burkholderiaceae |  |
| K11898 | type VI secretion system protein ImpE | Proteobacteria | Betaproteobacteria | Burkholderiales | Comamonadaceae |  |
| K11898 | type VI secretion system protein ImpE | Proteobacteria | Betaproteobacteria | Burkholderiales | Comamonadaceae | Variovorax |
| K11898 | type VI secretion system protein ImpE | Proteobacteria | Betaproteobacteria | Burkholderiales |  |  |
| K11898 | type VI secretion system protein ImpE | Proteobacteria | Gammaproteobacteria | Pseudomonadales | Pseudomonadaceae | Pseudomonas |
| K11898 | type VI secretion system protein ImpE | Verrucomicrobia | Verrucomicrobiae | Verrucomicrobiales | Verrucomicrobiaceae | Haloferula |
| K11897 | type VI secretion system protein ImpF | Proteobacteria | Alphaproteobacteria | Rhizobiales |  |  |
| K11897 | type VI secretion system protein ImpF | Proteobacteria | Alphaproteobacteria | Rhizobiales | Hyphomicrobiaceae | Devosia |
| K11897 | type VI secretion system protein ImpF | Proteobacteria | Alphaproteobacteria | Rhizobiales | Rhizobiaceae |  |
| K11897 | type VI secretion system protein ImpF | Proteobacteria | Alphaproteobacteria | Rhodobacterales | Rhodobacteraceae |  |
| K11897 | type VI secretion system protein ImpF | Proteobacteria | Alphaproteobacteria | Rhodospirillales |  |  |
| K11897 | type VI secretion system protein ImpF | Proteobacteria | Alphaproteobacteria | Sphingomonadales | Erythrobacteraceae |  |
| K11897 | type VI secretion system protein ImpF | Proteobacteria | Betaproteobacteria | Burkholderiales |  |  |
| K11897 | type VI secretion system protein ImpF | Proteobacteria | Betaproteobacteria | Burkholderiales | Comamonadaceae |  |
| K11897 | type VI secretion system protein ImpF | Proteobacteria | Betaproteobacteria | Burkholderiales | Comamonadaceae | Variovorax |
| K11897 | type VI secretion system protein ImpF | Proteobacteria | Betaproteobacteria | Burkholderiales | Oxalobacteraceae |  |
| K11897 | type VI secretion system protein ImpF | Proteobacteria | Betaproteobacteria | Burkholderiales | Oxalobacteraceae | Massilia |
| K11897 | type VI secretion system protein ImpF | Proteobacteria | Betaproteobacteria | Burkholderiales |  |  |
| K11897 | type VI secretion system protein ImpF | Proteobacteria | Deltaproteobacteria | Myxococcales | Sorangiineae | Polyangiaceae |
| K11897 | type VI secretion system protein ImpF | Proteobacteria | Gammaproteobacteria | Pseudomonadales | Pseudomonadaceae | Pseudomonas |
| K11896 | type VI secretion system protein ImpG | Acidobacteria |  |  |  |  |
| K11896 | type VI secretion system protein ImpG | Bacteroidetes |  |  |  |  |
| K11896 | type VI secretion system protein ImpG | Planctomycetes | Planctomycetia | Planctomycetales | Gemmataceae |  |
| K11896 | type VI secretion system protein ImpG | Planctomycetes | Planctomycetia | Planctomycetales | Planctomycetaceae |  |
| K11896 | type VI secretion system protein ImpG | Proteobacteria | Alphaproteobacteria | Rhizobiales | Phyllobacteriaceae |  |
| K11896 | type VI secretion system protein ImpG | Proteobacteria | Alphaproteobacteria | Rhizobiales | Phyllobacteriaceae | Mesorhizobium |
| K11896 | type VI secretion system protein ImpG | Proteobacteria | Alphaproteobacteria | Rhizobiales | Rhizobiaceae |  |
| K11896 | type VI secretion system protein ImpG | Proteobacteria | Alphaproteobacteria | Rhizobiales | Rhizobiaceae | Rhizobium/Agrobacterium group |
| K11896 | type VI secretion system protein ImpG | Proteobacteria | Alphaproteobacteria | Rhodobacterales | Rhodobacteraceae |  |
| K11896 | type VI secretion system protein ImpG | Proteobacteria | Alphaproteobacteria | Rhodospirillales |  |  |
| K11896 | type VI secretion system protein ImpG | Proteobacteria | Alphaproteobacteria | Sphingomonadales | Erythrobacteraceae |  |
| K11896 | type VI secretion system protein ImpG | Proteobacteria | Betaproteobacteria | Burkholderiales | Alcaligenaceae |  |
| K11896 | type VI secretion system protein ImpG | Proteobacteria | Betaproteobacteria | Burkholderiales | Burkholderiaceae |  |
| K11896 | type VI secretion system protein ImpG | Proteobacteria | Betaproteobacteria | Burkholderiales | Comamonadaceae |  |
| K11896 | type VI secretion system protein ImpG | Proteobacteria | Betaproteobacteria | Burkholderiales | Comamonadaceae | Variovorax |
| K11896 | type VI secretion system protein ImpG | Proteobacteria | Betaproteobacteria | Burkholderiales | Oxalobacteraceae |  |
| K11896 | type VI secretion system protein ImpG | Proteobacteria | Betaproteobacteria | Burkholderiales | Oxalobacteraceae | Massilia |
| K11896 | type VI secretion system protein ImpG | Proteobacteria | Betaproteobacteria | Burkholderiales |  |  |
| K11896 | type VI secretion system protein ImpG | Proteobacteria | Betaproteobacteria | Burkholderiales |  |  |
| K11896 | type VI secretion system protein ImpG | Proteobacteria | Deltaproteobacteria | Myxococcales | Cystobacterineae |  |
| K11896 | type VI secretion system protein ImpG | Proteobacteria | Deltaproteobacteria | Myxococcales | Cystobacterineae | Archangiaceae |
| K11896 | type VI secretion system protein ImpG | Proteobacteria | Deltaproteobacteria | Myxococcales | Cystobacterineae | Myxococcaceae |
| K11896 | type VI secretion system protein ImpG | Proteobacteria | Deltaproteobacteria | Myxococcales | Nannocystineae |  |
| K11896 | type VI secretion system protein ImpG | Proteobacteria | Deltaproteobacteria | Myxococcales | Nannocystineae | Nannocystaceae |
| K11896 | type VI secretion system protein ImpG | Proteobacteria | Deltaproteobacteria | Myxococcales | Sorangiineae |  |
| K11896 | type VI secretion system protein ImpG | Proteobacteria | Deltaproteobacteria | Myxococcales | Sorangiineae | Polyangiaceae |
| K11896 | type VI secretion system protein ImpG | Proteobacteria | Deltaproteobacteria | Myxococcales | Sorangiineae | Polyangiaceae |
| K11896 | type VI secretion system protein ImpG | Proteobacteria | Deltaproteobacteria | Myxococcales | Sorangiineae | Sandaracinaceae |
| K11896 | type VI secretion system protein ImpG | Proteobacteria | Deltaproteobacteria | Myxococcales |  |  |
| K11896 | type VI secretion system protein ImpG | Proteobacteria | Gammaproteobacteria | Cellvibrionales | Cellvibrionaceae | Cellvibrio |
| K11896 | type VI secretion system protein ImpG | Proteobacteria | Gammaproteobacteria | Pseudomonadales | Pseudomonadaceae |  |
| K11896 | type VI secretion system protein ImpG | Proteobacteria | Gammaproteobacteria | Pseudomonadales | Pseudomonadaceae | Pseudomonas |
| K11896 | type VI secretion system protein ImpG | Proteobacteria | Gammaproteobacteria | Xanthomonadales | Xanthomonadaceae |  |
| K11896 | type VI secretion system protein ImpG | Proteobacteria | Gammaproteobacteria | Xanthomonadales | Xanthomonadaceae | Lysobacter |
| K11896 | type VI secretion system protein ImpG | Verrucomicrobia | Verrucomicrobiae | Verrucomicrobiales | Verrucomicrobiaceae | Haloferula |
| K11895 | type VI secretion system protein ImpH | Acidobacteria |  |  |  |  |
| K11895 | type VI secretion system protein ImpH | Gemmatimonadetes |  |  |  |  |
| K11895 | type VI secretion system protein ImpH | Proteobacteria | Alphaproteobacteria | Rhizobiales | Hyphomicrobiaceae | Devosia |
| K11895 | type VI secretion system protein ImpH | Proteobacteria | Alphaproteobacteria | Rhizobiales | Phyllobacteriaceae |  |
| K11895 | type VI secretion system protein ImpH | Proteobacteria | Alphaproteobacteria | Rhizobiales | Phyllobacteriaceae | Mesorhizobium |
| K11895 | type VI secretion system protein ImpH | Proteobacteria | Alphaproteobacteria | Rhizobiales | Rhizobiaceae |  |
| K11895 | type VI secretion system protein ImpH | Proteobacteria | Alphaproteobacteria | Rhizobiales | Rhizobiaceae | Rhizobium/Agrobacterium group |
| K11895 | type VI secretion system protein ImpH | Proteobacteria | Alphaproteobacteria | Rhizobiales | Rhizobiaceae | Sinorhizobium/Ensifer group |
| K11895 | type VI secretion system protein ImpH | Proteobacteria | Alphaproteobacteria | Rhodobacterales | Rhodobacteraceae |  |
| K11895 | type VI secretion system protein ImpH | Proteobacteria | Alphaproteobacteria | Sphingomonadales | Erythrobacteraceae |  |
| K11895 | type VI secretion system protein ImpH | Proteobacteria | Betaproteobacteria | Burkholderiales | Comamonadaceae |  |
| K11895 | type VI secretion system protein ImpH | Proteobacteria | Betaproteobacteria | Burkholderiales | Comamonadaceae | Variovorax |
| K11895 | type VI secretion system protein ImpH | Proteobacteria | Betaproteobacteria | Burkholderiales | Oxalobacteraceae |  |
| K11895 | type VI secretion system protein ImpH | Proteobacteria | Betaproteobacteria | Burkholderiales | Oxalobacteraceae | Massilia |
| K11895 | type VI secretion system protein ImpH | Proteobacteria | Deltaproteobacteria | Myxococcales | Cystobacterineae |  |
| K11895 | type VI secretion system protein ImpH | Proteobacteria | Deltaproteobacteria | Myxococcales | Cystobacterineae | Archangiaceae |
| K11895 | type VI secretion system protein ImpH | Proteobacteria | Deltaproteobacteria | Myxococcales | Cystobacterineae | Myxococcaceae |
| K11895 | type VI secretion system protein ImpH | Proteobacteria | Deltaproteobacteria | Myxococcales | Nannocystineae | Nannocystaceae |
| K11895 | type VI secretion system protein ImpH | Proteobacteria | Deltaproteobacteria | Myxococcales | Sorangiineae |  |
| K11895 | type VI secretion system protein ImpH | Proteobacteria | Deltaproteobacteria | Myxococcales | Sorangiineae | Polyangiaceae |
| K11895 | type VI secretion system protein ImpH | Proteobacteria | Deltaproteobacteria | Myxococcales | Sorangiineae | Polyangiaceae |
| K11895 | type VI secretion system protein ImpH | Proteobacteria | Deltaproteobacteria | Myxococcales | Sorangiineae | Sandaracinaceae |
| K11895 | type VI secretion system protein ImpH | Proteobacteria | Gammaproteobacteria | Cellvibrionales | Cellvibrionaceae |  |
| K11895 | type VI secretion system protein ImpH | Proteobacteria | Gammaproteobacteria | Cellvibrionales | Cellvibrionaceae | Cellvibrio |
| K11895 | type VI secretion system protein ImpH | Proteobacteria | Gammaproteobacteria | Pseudomonadales | Pseudomonadaceae | Pseudomonas |
| K11895 | type VI secretion system protein ImpH | Proteobacteria | Gammaproteobacteria | Xanthomonadales | Xanthomonadaceae | Lysobacter |
| K11895 | type VI secretion system protein ImpH | Verrucomicrobia | Verrucomicrobiae | Verrucomicrobiales | Verrucomicrobiaceae | Haloferula |
| K11894 | type VI secretion system protein ImpI | Proteobacteria | Alphaproteobacteria | Rhizobiales | Rhizobiaceae |  |
| K11894 | type VI secretion system protein ImpI | Proteobacteria | Alphaproteobacteria | Rhizobiales | Rhizobiaceae | Sinorhizobium/Ensifer group |
| K11894 | type VI secretion system protein ImpI | Proteobacteria | Alphaproteobacteria | Rhodospirillales |  |  |
| K11894 | type VI secretion system protein ImpI | Proteobacteria | Deltaproteobacteria | Myxococcales | Sorangiineae |  |
| K11894 | type VI secretion system protein ImpI | Proteobacteria | Deltaproteobacteria | Myxococcales | Sorangiineae | Polyangiaceae |
| K11894 | type VI secretion system protein ImpI | Proteobacteria | Deltaproteobacteria | Myxococcales | Sorangiineae | Polyangiaceae |
| K11894 | type VI secretion system protein ImpI | Proteobacteria | Deltaproteobacteria | Myxococcales | Sorangiineae | Sandaracinaceae |
| K11894 | type VI secretion system protein ImpI | Proteobacteria | Gammaproteobacteria | Pseudomonadales | Pseudomonadaceae | Pseudomonas |
| K11893 | type VI secretion system protein ImpJ | Gemmatimonadetes |  |  |  |  |
| K11893 | type VI secretion system protein ImpJ | Planctomycetes | Planctomycetia | Planctomycetales |  |  |
| K11893 | type VI secretion system protein ImpJ | Planctomycetes | Planctomycetia | Planctomycetales | Planctomycetaceae |  |
| K11893 | type VI secretion system protein ImpJ | Proteobacteria | Alphaproteobacteria |  |  |  |
| K11893 | type VI secretion system protein ImpJ | Proteobacteria | Alphaproteobacteria | Rhizobiales |  |  |
| K11893 | type VI secretion system protein ImpJ | Proteobacteria | Alphaproteobacteria | Rhizobiales | Phyllobacteriaceae |  |
| K11893 | type VI secretion system protein ImpJ | Proteobacteria | Alphaproteobacteria | Rhizobiales | Phyllobacteriaceae | Mesorhizobium |
| K11893 | type VI secretion system protein ImpJ | Proteobacteria | Alphaproteobacteria | Rhizobiales | Rhizobiaceae |  |
| K11893 | type VI secretion system protein ImpJ | Proteobacteria | Alphaproteobacteria | Rhizobiales | Rhizobiaceae | Rhizobium/Agrobacterium group |
| K11893 | type VI secretion system protein ImpJ | Proteobacteria | Alphaproteobacteria | Rhizobiales | Rhizobiaceae | Rhizobium/Agrobacterium group |
| K11893 | type VI secretion system protein ImpJ | Proteobacteria | Alphaproteobacteria | Rhodobacterales | Rhodobacteraceae |  |
| K11893 | type VI secretion system protein ImpJ | Proteobacteria | Alphaproteobacteria | Rhodospirillales |  |  |
| K11893 | type VI secretion system protein ImpJ | Proteobacteria | Alphaproteobacteria | Rhodospirillales | Rhodospirillaceae |  |
| K11893 | type VI secretion system protein ImpJ | Proteobacteria | Alphaproteobacteria | Sphingomonadales | Erythrobacteraceae |  |
| K11893 | type VI secretion system protein ImpJ | Proteobacteria | Betaproteobacteria | Burkholderiales | Alcaligenaceae |  |
| K11893 | type VI secretion system protein ImpJ | Proteobacteria | Betaproteobacteria | Burkholderiales | Burkholderiaceae |  |
| K11893 | type VI secretion system protein ImpJ | Proteobacteria | Betaproteobacteria | Burkholderiales | Comamonadaceae |  |
| K11893 | type VI secretion system protein ImpJ | Proteobacteria | Betaproteobacteria | Burkholderiales | Comamonadaceae | Variovorax |
| K11893 | type VI secretion system protein ImpJ | Proteobacteria | Betaproteobacteria | Burkholderiales | Oxalobacteraceae |  |
| K11893 | type VI secretion system protein ImpJ | Proteobacteria | Betaproteobacteria | Burkholderiales | Oxalobacteraceae | Massilia |
| K11893 | type VI secretion system protein ImpJ | Proteobacteria | Betaproteobacteria | Burkholderiales |  |  |
| K11893 | type VI secretion system protein ImpJ | Proteobacteria | Deltaproteobacteria | Myxococcales |  |  |
| K11893 | type VI secretion system protein ImpJ | Proteobacteria | Deltaproteobacteria | Myxococcales | Cystobacterineae |  |
| K11893 | type VI secretion system protein ImpJ | Proteobacteria | Deltaproteobacteria | Myxococcales | Cystobacterineae | Archangiaceae |
| K11893 | type VI secretion system protein ImpJ | Proteobacteria | Deltaproteobacteria | Myxococcales | Cystobacterineae | Myxococcaceae |
| K11893 | type VI secretion system protein ImpJ | Proteobacteria | Deltaproteobacteria | Myxococcales | Nannocystineae |  |
| K11893 | type VI secretion system protein ImpJ | Proteobacteria | Deltaproteobacteria | Myxococcales | Nannocystineae | Nannocystaceae |
| K11893 | type VI secretion system protein ImpJ | Proteobacteria | Deltaproteobacteria | Myxococcales | Sorangiineae |  |
| K11893 | type VI secretion system protein ImpJ | Proteobacteria | Deltaproteobacteria | Myxococcales | Sorangiineae | Polyangiaceae |
| K11893 | type VI secretion system protein ImpJ | Proteobacteria | Deltaproteobacteria | Myxococcales | Sorangiineae | Polyangiaceae |
| K11893 | type VI secretion system protein ImpJ | Proteobacteria | Deltaproteobacteria | Myxococcales | Sorangiineae | Sandaracinaceae |
| K11893 | type VI secretion system protein ImpJ | Proteobacteria | Deltaproteobacteria | Myxococcales |  |  |
| K11893 | type VI secretion system protein ImpJ | Proteobacteria | Gammaproteobacteria | Pseudomonadales | Pseudomonadaceae |  |
| K11893 | type VI secretion system protein ImpJ | Proteobacteria | Gammaproteobacteria | Pseudomonadales | Pseudomonadaceae | Pseudomonas |
| K11893 | type VI secretion system protein ImpJ | Proteobacteria | Gammaproteobacteria | Xanthomonadales |  |  |
| K11893 | type VI secretion system protein ImpJ | Proteobacteria | Gammaproteobacteria | Xanthomonadales | Xanthomonadaceae | Lysobacter |
| K11892 | type VI secretion system protein ImpK | Acidobacteria | unclassified Acidobacteria | Acidobacteria subdivision 6 | Vicinamibacteraceae | Luteitalea |
| K11892 | type VI secretion system protein ImpK | Planctomycetes | Planctomycetia | Planctomycetales | Planctomycetaceae |  |
| K11892 | type VI secretion system protein ImpK | Proteobacteria | Alphaproteobacteria |  |  |  |
| K11892 | type VI secretion system protein ImpK | Proteobacteria | Alphaproteobacteria | Rhizobiales |  |  |
| K11892 | type VI secretion system protein ImpK | Proteobacteria | Alphaproteobacteria | Rhizobiales | Phyllobacteriaceae |  |
| K11892 | type VI secretion system protein ImpK | Proteobacteria | Alphaproteobacteria | Rhizobiales | Phyllobacteriaceae | Mesorhizobium |
| K11892 | type VI secretion system protein ImpK | Proteobacteria | Alphaproteobacteria | Rhizobiales | Rhizobiaceae |  |
| K11892 | type VI secretion system protein ImpK | Proteobacteria | Alphaproteobacteria | Rhizobiales | Rhizobiaceae | Rhizobium/Agrobacterium group |
| K11892 | type VI secretion system protein ImpK | Proteobacteria | Alphaproteobacteria | Rhodobacterales | Rhodobacteraceae |  |
| K11892 | type VI secretion system protein ImpK | Proteobacteria | Alphaproteobacteria | Rhodospirillales |  |  |
| K11892 | type VI secretion system protein ImpK | Proteobacteria | Alphaproteobacteria | Rhodospirillales | Rhodospirillaceae |  |
| K11892 | type VI secretion system protein ImpK | Proteobacteria | Alphaproteobacteria | Sphingomonadales |  |  |
| K11892 | type VI secretion system protein ImpK | Proteobacteria | Alphaproteobacteria | Sphingomonadales | Erythrobacteraceae |  |
| K11892 | type VI secretion system protein ImpK | Proteobacteria | Betaproteobacteria | Burkholderiales |  |  |
| K11892 | type VI secretion system protein ImpK | Proteobacteria | Betaproteobacteria | Burkholderiales | Alcaligenaceae |  |
| K11892 | type VI secretion system protein ImpK | Proteobacteria | Betaproteobacteria | Burkholderiales | Burkholderiaceae |  |
| K11892 | type VI secretion system protein ImpK | Proteobacteria | Betaproteobacteria | Burkholderiales | Comamonadaceae |  |
| K11892 | type VI secretion system protein ImpK | Proteobacteria | Betaproteobacteria | Burkholderiales | Comamonadaceae | Acidovorax |
| K11892 | type VI secretion system protein ImpK | Proteobacteria | Betaproteobacteria | Burkholderiales | Comamonadaceae | Variovorax |
| K11892 | type VI secretion system protein ImpK | Proteobacteria | Betaproteobacteria | Burkholderiales | Oxalobacteraceae |  |
| K11892 | type VI secretion system protein ImpK | Proteobacteria | Betaproteobacteria | Burkholderiales | Oxalobacteraceae | Massilia |
| K11892 | type VI secretion system protein ImpK | Proteobacteria | Betaproteobacteria | Burkholderiales |  |  |
| K11892 | type VI secretion system protein ImpK | Proteobacteria | Deltaproteobacteria | Myxococcales | Cystobacterineae |  |
| K11892 | type VI secretion system protein ImpK | Proteobacteria | Deltaproteobacteria | Myxococcales | Sorangiineae |  |
| K11892 | type VI secretion system protein ImpK | Proteobacteria | Deltaproteobacteria | Myxococcales | Sorangiineae | Polyangiaceae |
| K11892 | type VI secretion system protein ImpK | Proteobacteria | Deltaproteobacteria | Myxococcales | Sorangiineae | Polyangiaceae |
| K11892 | type VI secretion system protein ImpK | Proteobacteria | Deltaproteobacteria | Myxococcales | Sorangiineae | Sandaracinaceae |
| K11892 | type VI secretion system protein ImpK | Proteobacteria | Gammaproteobacteria | Cellvibrionales | Cellvibrionaceae | Cellvibrio |
| K11892 | type VI secretion system protein ImpK | Proteobacteria | Gammaproteobacteria | Pseudomonadales | Pseudomonadaceae | Pseudomonas |
| K11892 | type VI secretion system protein ImpK | Proteobacteria | Gammaproteobacteria | Xanthomonadales | Xanthomonadaceae |  |
| K11891 | type VI secretion system protein ImpL | Acidobacteria |  |  |  |  |
| K11891 | type VI secretion system protein ImpL | Gemmatimonadetes |  |  |  |  |
| K11891 | type VI secretion system protein ImpL | Proteobacteria | Alphaproteobacteria | Rhizobiales |  |  |
| K11891 | type VI secretion system protein ImpL | Proteobacteria | Alphaproteobacteria | Rhizobiales | Hyphomicrobiaceae | Devosia |
| K11891 | type VI secretion system protein ImpL | Proteobacteria | Alphaproteobacteria | Rhizobiales | Phyllobacteriaceae |  |
| K11891 | type VI secretion system protein ImpL | Proteobacteria | Alphaproteobacteria | Rhizobiales | Phyllobacteriaceae | Mesorhizobium |
| K11891 | type VI secretion system protein ImpL | Proteobacteria | Alphaproteobacteria | Rhizobiales | Rhizobiaceae |  |
| K11891 | type VI secretion system protein ImpL | Proteobacteria | Alphaproteobacteria | Rhizobiales | Rhizobiaceae | Rhizobium/Agrobacterium group |
| K11891 | type VI secretion system protein ImpL | Proteobacteria | Alphaproteobacteria | Rhizobiales | Rhizobiaceae | Rhizobium/Agrobacterium group |
| K11891 | type VI secretion system protein ImpL | Proteobacteria | Alphaproteobacteria | Rhizobiales |  |  |
| K11891 | type VI secretion system protein ImpL | Proteobacteria | Alphaproteobacteria | Rhodobacterales | Rhodobacteraceae |  |
| K11891 | type VI secretion system protein ImpL | Proteobacteria | Alphaproteobacteria | Rhodospirillales |  |  |
| K11891 | type VI secretion system protein ImpL | Proteobacteria | Alphaproteobacteria | Sphingomonadales | Erythrobacteraceae |  |
| K11891 | type VI secretion system protein ImpL | Proteobacteria | Alphaproteobacteria | Sphingomonadales | Sphingomonadaceae | Sphingomonas |
| K11891 | type VI secretion system protein ImpL | Proteobacteria | Betaproteobacteria | Burkholderiales |  |  |
| K11891 | type VI secretion system protein ImpL | Proteobacteria | Betaproteobacteria | Burkholderiales | Alcaligenaceae |  |
| K11891 | type VI secretion system protein ImpL | Proteobacteria | Betaproteobacteria | Burkholderiales | Burkholderiaceae |  |
| K11891 | type VI secretion system protein ImpL | Proteobacteria | Betaproteobacteria | Burkholderiales | Comamonadaceae |  |
| K11891 | type VI secretion system protein ImpL | Proteobacteria | Betaproteobacteria | Burkholderiales | Comamonadaceae | Variovorax |
| K11891 | type VI secretion system protein ImpL | Proteobacteria | Betaproteobacteria | Burkholderiales | Oxalobacteraceae |  |
| K11891 | type VI secretion system protein ImpL | Proteobacteria | Betaproteobacteria | Burkholderiales | Oxalobacteraceae | Massilia |
| K11891 | type VI secretion system protein ImpL | Proteobacteria | Betaproteobacteria | Burkholderiales |  |  |
| K11891 | type VI secretion system protein ImpL | Proteobacteria | Betaproteobacteria | Burkholderiales |  |  |
| K11891 | type VI secretion system protein ImpL | Proteobacteria | Betaproteobacteria | Nitrosomonadales |  |  |
| K11891 | type VI secretion system protein ImpL | Proteobacteria | Deltaproteobacteria | Myxococcales |  |  |
| K11891 | type VI secretion system protein ImpL | Proteobacteria | Deltaproteobacteria | Myxococcales | Cystobacterineae |  |
| K11891 | type VI secretion system protein ImpL | Proteobacteria | Deltaproteobacteria | Myxococcales | Cystobacterineae | Archangiaceae |
| K11891 | type VI secretion system protein ImpL | Proteobacteria | Deltaproteobacteria | Myxococcales | Cystobacterineae | Myxococcaceae |
| K11891 | type VI secretion system protein ImpL | Proteobacteria | Deltaproteobacteria | Myxococcales | Sorangiineae |  |
| K11891 | type VI secretion system protein ImpL | Proteobacteria | Deltaproteobacteria | Myxococcales | Sorangiineae | Polyangiaceae |
| K11891 | type VI secretion system protein ImpL | Proteobacteria | Deltaproteobacteria | Myxococcales | Sorangiineae | Polyangiaceae |
| K11891 | type VI secretion system protein ImpL | Proteobacteria | Deltaproteobacteria | Myxococcales | Sorangiineae | Sandaracinaceae |
| K11891 | type VI secretion system protein ImpL | Proteobacteria | Gammaproteobacteria | Cellvibrionales | Cellvibrionaceae | Cellvibrio |
| K11891 | type VI secretion system protein ImpL | Proteobacteria | Gammaproteobacteria | Pseudomonadales |  |  |
| K11891 | type VI secretion system protein ImpL | Proteobacteria | Gammaproteobacteria | Pseudomonadales | Pseudomonadaceae | Pseudomonas |
| K11891 | type VI secretion system protein ImpL | Proteobacteria | Gammaproteobacteria | Xanthomonadales |  |  |
| K11891 | type VI secretion system protein ImpL | Proteobacteria | Gammaproteobacteria | Xanthomonadales | Xanthomonadaceae | Lysobacter |
| K11890 | type VI secretion system protein ImpM | Proteobacteria | Alphaproteobacteria | Rhizobiales | Hyphomicrobiaceae | Devosia |
| K11890 | type VI secretion system protein ImpM | Proteobacteria | Alphaproteobacteria | Rhizobiales | Phyllobacteriaceae |  |
| K11890 | type VI secretion system protein ImpM | Proteobacteria | Alphaproteobacteria | Rhizobiales | Rhizobiaceae |  |
| K11890 | type VI secretion system protein ImpM | Proteobacteria | Alphaproteobacteria | Rhizobiales | Rhizobiaceae | Rhizobium/Agrobacterium group |
| K11890 | type VI secretion system protein ImpM | Proteobacteria | Alphaproteobacteria | Rhizobiales |  |  |
| K11890 | type VI secretion system protein ImpM | Proteobacteria | Alphaproteobacteria | Rhodobacterales | Rhodobacteraceae |  |
| K11890 | type VI secretion system protein ImpM | Proteobacteria | Alphaproteobacteria | Rhodobacterales | Rhodobacteraceae | Rubellimicrobium |
| K11890 | type VI secretion system protein ImpM | Proteobacteria | Alphaproteobacteria | Sphingomonadales |  |  |
| K11890 | type VI secretion system protein ImpM | Proteobacteria | Alphaproteobacteria | Sphingomonadales | Erythrobacteraceae |  |
| K11890 | type VI secretion system protein ImpM | Proteobacteria | Alphaproteobacteria | Sphingomonadales | Sphingomonadaceae | Sphingomonas |
| K11890 | type VI secretion system protein ImpM | Proteobacteria | Betaproteobacteria | Burkholderiales |  |  |
| K11890 | type VI secretion system protein ImpM | Proteobacteria | Betaproteobacteria | Burkholderiales | Comamonadaceae |  |
| K11890 | type VI secretion system protein ImpM | Proteobacteria | Betaproteobacteria | Burkholderiales | Comamonadaceae | Variovorax |
| K11890 | type VI secretion system protein ImpM | Proteobacteria | Betaproteobacteria | Burkholderiales | Oxalobacteraceae |  |
| K11890 | type VI secretion system protein ImpM | Proteobacteria | Betaproteobacteria | Burkholderiales | Oxalobacteraceae | Massilia |
| K11890 | type VI secretion system protein ImpM | Proteobacteria | Deltaproteobacteria | Myxococcales |  |  |
| K11890 | type VI secretion system protein ImpM | Proteobacteria | Deltaproteobacteria | Myxococcales | Cystobacterineae | Myxococcaceae |
| K11890 | type VI secretion system protein ImpM | Proteobacteria | Deltaproteobacteria | Myxococcales | Sorangiineae |  |
| K11890 | type VI secretion system protein ImpM | Proteobacteria | Deltaproteobacteria | Myxococcales | Sorangiineae | Polyangiaceae |
| K11890 | type VI secretion system protein ImpM | Proteobacteria | Deltaproteobacteria | Myxococcales | Sorangiineae | Polyangiaceae |
| K11890 | type VI secretion system protein ImpM | Proteobacteria | Deltaproteobacteria | Myxococcales | Sorangiineae | Sandaracinaceae |
| K11890 | type VI secretion system protein ImpM | Proteobacteria | Gammaproteobacteria | Cellvibrionales | Cellvibrionaceae | Cellvibrio |
| K11890 | type VI secretion system protein ImpM | Proteobacteria | Gammaproteobacteria | Nevskiales | Sinobacteraceae | Steroidobacter |
| K11890 | type VI secretion system protein ImpM | Proteobacteria | Gammaproteobacteria | Pseudomonadales | Pseudomonadaceae | Pseudomonas |
| K11890 | type VI secretion system protein ImpM | Proteobacteria | Gammaproteobacteria | Xanthomonadales | Xanthomonadaceae | Lysobacter |
| K11906 | type VI secretion system protein VasD | Proteobacteria | Alphaproteobacteria | Rhizobiales |  |  |
| K11906 | type VI secretion system protein VasD | Proteobacteria | Alphaproteobacteria | Rhizobiales | Phyllobacteriaceae |  |
| K11906 | type VI secretion system protein VasD | Proteobacteria | Alphaproteobacteria | Rhizobiales | Rhizobiaceae |  |
| K11906 | type VI secretion system protein VasD | Proteobacteria | Alphaproteobacteria | Rhizobiales | Rhizobiaceae | Rhizobium/Agrobacterium group |
| K11906 | type VI secretion system protein VasD | Proteobacteria | Alphaproteobacteria | Rhodobacterales | Rhodobacteraceae |  |
| K11906 | type VI secretion system protein VasD | Proteobacteria | Betaproteobacteria | Burkholderiales |  |  |
| K11906 | type VI secretion system protein VasD | Proteobacteria | Betaproteobacteria | Burkholderiales | Comamonadaceae |  |
| K11906 | type VI secretion system protein VasD | Proteobacteria | Betaproteobacteria | Burkholderiales | Comamonadaceae | Variovorax |
| K11906 | type VI secretion system protein VasD | Proteobacteria | Betaproteobacteria | Burkholderiales | Oxalobacteraceae |  |
| K11906 | type VI secretion system protein VasD | Proteobacteria | Betaproteobacteria | Burkholderiales |  |  |
| K11906 | type VI secretion system protein VasD | Proteobacteria | Deltaproteobacteria | Myxococcales |  |  |
| K11906 | type VI secretion system protein VasD | Proteobacteria | Deltaproteobacteria | Myxococcales | Cystobacterineae |  |
| K11906 | type VI secretion system protein VasD | Proteobacteria | Deltaproteobacteria | Myxococcales | Sorangiineae |  |
| K11906 | type VI secretion system protein VasD | Proteobacteria | Deltaproteobacteria | Myxococcales | Sorangiineae | Polyangiaceae |
| K11906 | type VI secretion system protein VasD | Proteobacteria | Deltaproteobacteria | Myxococcales | Sorangiineae | Sandaracinaceae |
| K11906 | type VI secretion system protein VasD | Proteobacteria | Gammaproteobacteria | Cellvibrionales | Cellvibrionaceae | Cellvibrio |
| K11906 | type VI secretion system protein VasD | Proteobacteria | Gammaproteobacteria | Pseudomonadales | Pseudomonadaceae | Pseudomonas |
| K11906 | type VI secretion system protein VasD | Proteobacteria | Gammaproteobacteria | Xanthomonadales | Xanthomonadaceae | Lysobacter |
| K11907 | type VI secretion system protein VasG | Acidobacteria |  |  |  |  |
| K11907 | type VI secretion system protein VasG | Proteobacteria | Alphaproteobacteria |  |  |  |
| K11907 | type VI secretion system protein VasG | Proteobacteria | Alphaproteobacteria | Rhizobiales |  |  |
| K11907 | type VI secretion system protein VasG | Proteobacteria | Alphaproteobacteria | Rhizobiales | Phyllobacteriaceae |  |
| K11907 | type VI secretion system protein VasG | Proteobacteria | Alphaproteobacteria | Rhizobiales | Phyllobacteriaceae | Mesorhizobium |
| K11907 | type VI secretion system protein VasG | Proteobacteria | Alphaproteobacteria | Rhizobiales | Rhizobiaceae |  |
| K11907 | type VI secretion system protein VasG | Proteobacteria | Alphaproteobacteria | Rhizobiales | Rhizobiaceae | Rhizobium/Agrobacterium group |
| K11907 | type VI secretion system protein VasG | Proteobacteria | Alphaproteobacteria | Rhizobiales | Rhizobiaceae | Rhizobium/Agrobacterium group |
| K11907 | type VI secretion system protein VasG | Proteobacteria | Alphaproteobacteria | Rhizobiales |  |  |
| K11907 | type VI secretion system protein VasG | Proteobacteria | Alphaproteobacteria | Rhodobacterales | Rhodobacteraceae |  |
| K11907 | type VI secretion system protein VasG | Proteobacteria | Alphaproteobacteria | Rhodospirillales | Rhodospirillaceae |  |
| K11907 | type VI secretion system protein VasG | Proteobacteria | Alphaproteobacteria | Sphingomonadales |  |  |
| K11907 | type VI secretion system protein VasG | Proteobacteria | Alphaproteobacteria | Sphingomonadales | Erythrobacteraceae |  |
| K11907 | type VI secretion system protein VasG | Proteobacteria | Betaproteobacteria | Burkholderiales |  |  |
| K11907 | type VI secretion system protein VasG | Proteobacteria | Betaproteobacteria | Burkholderiales | Burkholderiaceae |  |
| K11907 | type VI secretion system protein VasG | Proteobacteria | Betaproteobacteria | Burkholderiales | Comamonadaceae |  |
| K11907 | type VI secretion system protein VasG | Proteobacteria | Betaproteobacteria | Burkholderiales | Comamonadaceae | Acidovorax |
| K11907 | type VI secretion system protein VasG | Proteobacteria | Betaproteobacteria | Burkholderiales | Comamonadaceae | Variovorax |
| K11907 | type VI secretion system protein VasG | Proteobacteria | Betaproteobacteria | Burkholderiales | Oxalobacteraceae |  |
| K11907 | type VI secretion system protein VasG | Proteobacteria | Betaproteobacteria | Burkholderiales | Oxalobacteraceae | Massilia |
| K11907 | type VI secretion system protein VasG | Proteobacteria | Betaproteobacteria | Burkholderiales |  |  |
| K11907 | type VI secretion system protein VasG | Proteobacteria | Betaproteobacteria | Burkholderiales |  |  |
| K11907 | type VI secretion system protein VasG | Proteobacteria | Deltaproteobacteria | Myxococcales |  |  |
| K11907 | type VI secretion system protein VasG | Proteobacteria | Deltaproteobacteria | Myxococcales | Cystobacterineae |  |
| K11907 | type VI secretion system protein VasG | Proteobacteria | Deltaproteobacteria | Myxococcales | Cystobacterineae | Myxococcaceae |
| K11907 | type VI secretion system protein VasG | Proteobacteria | Deltaproteobacteria | Myxococcales | Sorangiineae |  |
| K11907 | type VI secretion system protein VasG | Proteobacteria | Deltaproteobacteria | Myxococcales | Sorangiineae | Polyangiaceae |
| K11907 | type VI secretion system protein VasG | Proteobacteria | Deltaproteobacteria | Myxococcales | Sorangiineae | Polyangiaceae |
| K11907 | type VI secretion system protein VasG | Proteobacteria | Deltaproteobacteria | Myxococcales | Sorangiineae | Sandaracinaceae |
| K11907 | type VI secretion system protein VasG | Proteobacteria | Gammaproteobacteria | Pseudomonadales |  |  |
| K11907 | type VI secretion system protein VasG | Proteobacteria | Gammaproteobacteria | Pseudomonadales | Pseudomonadaceae |  |
| K11907 | type VI secretion system protein VasG | Proteobacteria | Gammaproteobacteria | Pseudomonadales | Pseudomonadaceae | Pseudomonas |
| K11907 | type VI secretion system protein VasG | Proteobacteria | Gammaproteobacteria | Xanthomonadales | Xanthomonadaceae |  |
| K11907 | type VI secretion system protein VasG | Proteobacteria | Gammaproteobacteria | Xanthomonadales | Xanthomonadaceae | Lysobacter |
| K11907 | type VI secretion system protein VasG | Verrucomicrobia | Verrucomicrobiae | Verrucomicrobiales | Verrucomicrobiaceae | Haloferula |
| K11909 | type VI secretion system protein VasI | Proteobacteria | Betaproteobacteria |  |  |  |
| K11910 | type VI secretion system protein VasJ | Proteobacteria | Alphaproteobacteria | Rhizobiales | Rhizobiaceae | Rhizobium/Agrobacterium group |
| K11910 | type VI secretion system protein VasJ | Proteobacteria | Deltaproteobacteria | Myxococcales |  |  |
| K11910 | type VI secretion system protein VasJ | Proteobacteria | Deltaproteobacteria | Myxococcales | Cystobacterineae |  |
| K11910 | type VI secretion system protein VasJ | Proteobacteria | Deltaproteobacteria | Myxococcales | Cystobacterineae | Archangiaceae |
| K11910 | type VI secretion system protein VasJ | Proteobacteria | Deltaproteobacteria | Myxococcales | Cystobacterineae | Myxococcaceae |
| K11910 | type VI secretion system protein VasJ | Proteobacteria | Deltaproteobacteria | Myxococcales | Nannocystineae | Nannocystaceae |
| K11910 | type VI secretion system protein VasJ | Proteobacteria | Deltaproteobacteria | Myxococcales | Sorangiineae |  |
| K11910 | type VI secretion system protein VasJ | Proteobacteria | Deltaproteobacteria | Myxococcales | Sorangiineae | Polyangiaceae |
| K11910 | type VI secretion system protein VasJ | Proteobacteria | Deltaproteobacteria | Myxococcales | Sorangiineae | Polyangiaceae |
| K11910 | type VI secretion system protein VasJ | Proteobacteria | Gammaproteobacteria | Pseudomonadales | Pseudomonadaceae | Pseudomonas |
| K11903 | type VI secretion system secreted protein Hcp | Bacteroidetes | Cytophagia | Cytophagales |  |  |
| K11903 | type VI secretion system secreted protein Hcp | Planctomycetes | Planctomycetia | Planctomycetales |  |  |
| K11903 | type VI secretion system secreted protein Hcp | Planctomycetes | Planctomycetia | Planctomycetales | Planctomycetaceae |  |
| K11903 | type VI secretion system secreted protein Hcp | Proteobacteria | Alphaproteobacteria | Rhizobiales |  |  |
| K11903 | type VI secretion system secreted protein Hcp | Proteobacteria | Alphaproteobacteria | Rhizobiales | Rhizobiaceae |  |
| K11903 | type VI secretion system secreted protein Hcp | Proteobacteria | Alphaproteobacteria | Rhizobiales | Rhizobiaceae | Rhizobium/Agrobacterium group |
| K11903 | type VI secretion system secreted protein Hcp | Proteobacteria | Alphaproteobacteria | Rhodobacterales | Rhodobacteraceae |  |
| K11903 | type VI secretion system secreted protein Hcp | Proteobacteria | Alphaproteobacteria | Rhodospirillales |  |  |
| K11903 | type VI secretion system secreted protein Hcp | Proteobacteria | Alphaproteobacteria | Sphingomonadales | Erythrobacteraceae |  |
| K11903 | type VI secretion system secreted protein Hcp | Proteobacteria | Betaproteobacteria | Burkholderiales |  |  |
| K11903 | type VI secretion system secreted protein Hcp | Proteobacteria | Betaproteobacteria | Burkholderiales | Comamonadaceae |  |
| K11903 | type VI secretion system secreted protein Hcp | Proteobacteria | Betaproteobacteria | Burkholderiales | Comamonadaceae | Variovorax |
| K11903 | type VI secretion system secreted protein Hcp | Proteobacteria | Betaproteobacteria | Burkholderiales | Oxalobacteraceae |  |
| K11903 | type VI secretion system secreted protein Hcp | Proteobacteria | Betaproteobacteria | Burkholderiales |  |  |
| K11903 | type VI secretion system secreted protein Hcp | Proteobacteria | Deltaproteobacteria | Myxococcales |  |  |
| K11903 | type VI secretion system secreted protein Hcp | Proteobacteria | Deltaproteobacteria | Myxococcales | Cystobacterineae |  |
| K11903 | type VI secretion system secreted protein Hcp | Proteobacteria | Deltaproteobacteria | Myxococcales | Sorangiineae |  |
| K11903 | type VI secretion system secreted protein Hcp | Proteobacteria | Deltaproteobacteria | Myxococcales | Sorangiineae | Polyangiaceae |
| K11903 | type VI secretion system secreted protein Hcp | Proteobacteria | Deltaproteobacteria | Myxococcales | Sorangiineae | Sandaracinaceae |
| K11903 | type VI secretion system secreted protein Hcp | Proteobacteria | Gammaproteobacteria | Pseudomonadales |  |  |
| K11903 | type VI secretion system secreted protein Hcp | Proteobacteria | Gammaproteobacteria | Pseudomonadales | Pseudomonadaceae |  |
| K11903 | type VI secretion system secreted protein Hcp | Proteobacteria | Gammaproteobacteria | Pseudomonadales | Pseudomonadaceae | Pseudomonas |
| K11903 | type VI secretion system secreted protein Hcp | Proteobacteria | Gammaproteobacteria | Xanthomonadales | Xanthomonadaceae |  |
| K11903 | type VI secretion system secreted protein Hcp | Verrucomicrobia | Verrucomicrobiae | Verrucomicrobiales | Verrucomicrobiaceae | Haloferula |
| K11904 | type VI secretion system secreted protein VgrG | Planctomycetes | Planctomycetia | Planctomycetales |  |  |
| K11904 | type VI secretion system secreted protein VgrG | Proteobacteria | Alphaproteobacteria | Caulobacterales | Caulobacteraceae | Caulobacter |
| K11904 | type VI secretion system secreted protein VgrG | Proteobacteria | Alphaproteobacteria | Rhizobiales |  |  |
| K11904 | type VI secretion system secreted protein VgrG | Proteobacteria | Alphaproteobacteria | Rhizobiales | Phyllobacteriaceae |  |
| K11904 | type VI secretion system secreted protein VgrG | Proteobacteria | Alphaproteobacteria | Rhizobiales | Rhizobiaceae |  |
| K11904 | type VI secretion system secreted protein VgrG | Proteobacteria | Alphaproteobacteria | Rhizobiales | Rhizobiaceae | Rhizobium/Agrobacterium group |
| K11904 | type VI secretion system secreted protein VgrG | Proteobacteria | Alphaproteobacteria | Rhizobiales | Rhizobiaceae | Sinorhizobium/Ensifer group |
| K11904 | type VI secretion system secreted protein VgrG | Proteobacteria | Alphaproteobacteria | Rhodobacterales | Rhodobacteraceae |  |
| K11904 | type VI secretion system secreted protein VgrG | Proteobacteria | Alphaproteobacteria | Rhodospirillales |  |  |
| K11904 | type VI secretion system secreted protein VgrG | Proteobacteria | Alphaproteobacteria | Sphingomonadales | Erythrobacteraceae |  |
| K11904 | type VI secretion system secreted protein VgrG | Proteobacteria | Betaproteobacteria | Burkholderiales | Burkholderiaceae |  |
| K11904 | type VI secretion system secreted protein VgrG | Proteobacteria | Betaproteobacteria | Burkholderiales | Comamonadaceae |  |
| K11904 | type VI secretion system secreted protein VgrG | Proteobacteria | Betaproteobacteria | Burkholderiales | Comamonadaceae | Acidovorax |
| K11904 | type VI secretion system secreted protein VgrG | Proteobacteria | Betaproteobacteria | Burkholderiales | Comamonadaceae | Variovorax |
| K11904 | type VI secretion system secreted protein VgrG | Proteobacteria | Betaproteobacteria | Burkholderiales | Oxalobacteraceae |  |
| K11904 | type VI secretion system secreted protein VgrG | Proteobacteria | Betaproteobacteria | Burkholderiales | Oxalobacteraceae | Massilia |
| K11904 | type VI secretion system secreted protein VgrG | Proteobacteria | Deltaproteobacteria | Myxococcales | Cystobacterineae |  |
| K11904 | type VI secretion system secreted protein VgrG | Proteobacteria | Deltaproteobacteria | Myxococcales | Cystobacterineae | Archangiaceae |
| K11904 | type VI secretion system secreted protein VgrG | Proteobacteria | Deltaproteobacteria | Myxococcales | Cystobacterineae | Myxococcaceae |
| K11904 | type VI secretion system secreted protein VgrG | Proteobacteria | Deltaproteobacteria | Myxococcales | Nannocystineae | Nannocystaceae |
| K11904 | type VI secretion system secreted protein VgrG | Proteobacteria | Deltaproteobacteria | Myxococcales | Sorangiineae |  |
| K11904 | type VI secretion system secreted protein VgrG | Proteobacteria | Deltaproteobacteria | Myxococcales | Sorangiineae | Polyangiaceae |
| K11904 | type VI secretion system secreted protein VgrG | Proteobacteria | Deltaproteobacteria | Myxococcales | Sorangiineae | Polyangiaceae |
| K11904 | type VI secretion system secreted protein VgrG | Proteobacteria | Deltaproteobacteria | Myxococcales | Sorangiineae | Sandaracinaceae |
| K11904 | type VI secretion system secreted protein VgrG | Proteobacteria | Gammaproteobacteria | Cellvibrionales | Cellvibrionaceae | Cellvibrio |
| K11904 | type VI secretion system secreted protein VgrG | Proteobacteria | Gammaproteobacteria | Pseudomonadales | Pseudomonadaceae | Pseudomonas |
| K11904 | type VI secretion system secreted protein VgrG | Proteobacteria | Gammaproteobacteria | Xanthomonadales | Xanthomonadaceae | Lysobacter |
| K11904 | type VI secretion system secreted protein VgrG | Verrucomicrobia | Verrucomicrobiae | Verrucomicrobiales | Verrucomicrobiaceae | Haloferula |

**Table S6** Changes in soil and plant parameters as influenced by nitrate and CO_2_ levels in 6-week-old wheat (Includes all measured parameters per pot).

| Plant growth cycle | pot number | Nitrate (ppm) | CO_2_ (ppm) | Dry root weight (gr) | Dry leave weight (gr) | Soil moisture % | pH | EC uS/m2 in 5 gr dry soil | N-NO3 (mg/kg) dry soil | N-NH4 (mg/kg) dry soil | Total N in shoots (mg/kg) | Total N in root (mg/kg) | Total N in shoots (mg) | Total N in root (mg) |
| --- | --- | --- | --- | --- | --- | --- | --- | --- | --- | --- | --- | --- | --- | --- |
| 1 | 1 | 30 | 400 | 0.45 | 0.94 | 5.49 | 7.17 | 126 | 0.10 | 0.96 | 2107 | 1854 | 1.98 | 0.83 |
| 1 | 2 | 30 | 400 | 0.28 | 0.98 | 5.04 | 7.09 | 136 | 0.17 | 0.93 | 2064 | 3431 | 2.02 | 0.96 |
| 1 | 3 | 30 | 400 | 0.41 | 1.03 | 5.93 | 7.15 | 149 | 0.02 | 0.78 | 1746 | 1996 | 1.80 | 0.82 |
| 1 | 19 | 30 | 400 |  | 0.57 | 8.70 | 6.75 | 140 | 0.30 | 0.90 | 5621 |  | 3.20 |  |
| 1 | 20 | 30 | 400 |  | 0.53 | 10.13 | 6.45 | 228 | 0.67 | 0.83 | 5834 |  | 3.09 |  |
| 1 | 6 | 70 | 400 |  | 0.43 | 5.49 | 7.14 | 148 | 9.27 | 0.81 | 7721 |  | 3.32 |  |
| 1 | 7 | 70 | 400 | 0.05 | 1.16 | 6.16 | 6.42 | 212 | 17.12 | 0.62 | 3042 |  | 3.53 |  |
| 1 | 8 | 70 | 400 |  | 0.6 | 4.82 | 6.89 | 160 | 7.10 | 0.91 | 6790 |  | 4.07 |  |
| 1 | 24 | 70 | 400 | 0.05 |  | 4.17 | 6.77 | 97 | 0.05 | 0.87 |  | 26433 |  | 1.32 |
| 1 | 25 | 70 | 400 | 0.19 |  | 2.04 | 6.78 | 132 | 0.08 | 0.82 |  | 6392 |  | 1.21 |
| 1 | 11 | 100 | 400 |  | 0.58 | 9.41 | 7.08 | 127 | 4.57 | 0.83 | 6437 |  | 3.73 |  |
| 1 | 12 | 100 | 400 |  | 0.51 | 14.94 | 6.67 | 148 | 11.60 | 0.75 | 7020 |  | 3.58 |  |
| 1 | 13 | 100 | 400 |  | 0.57 | 13.38 | 6.57 | 146 | 12.80 | 0.89 | 6729 |  | 3.84 |  |
| 1 | 29 | 100 | 400 | 0.1 | 0.72 | 2.88 | 6.45 | 125 | 2.51 | 0.77 | 4524 |  | 3.26 |  |
| 1 | 30 | 100 | 400 | 0.12 | 0.75 | 5.26 | 6.51 | 118 | 6.66 | 0.75 | 4540 | 14633 | 3.40 | 1.76 |
| 1 | 31 | 30 | 850 | 0.35 | 1.31 | 4.82 | 6.05 | 114 | 0.04 | 0.72 | 1042 | 2594 | 1.37 | 0.91 |
| 1 | 32 | 30 | 850 | 0.58 | 1.84 | 3.52 | 6.52 | 131 | 0.04 | 0.73 | 760 | 1245 | 1.40 | 0.72 |
| 1 | 33 | 30 | 850 | 0.68 | 1.35 | 14.68 | 7.33 | 203 | 1.83 | 8.21 | 902 | 916 | 1.22 | 0.62 |
| 1 | 34 | 30 | 850 | 0.6 | 1.32 | 6.84 | 6.73 | 124 | 0.31 | 0.99 | 1134 | 1244 | 1.50 | 0.75 |
| 1 | 35 | 30 | 850 | 0.79 | 1.28 | 3.52 | 6.64 | 113 | 0.09 | 0.81 | 1076 | 776 | 1.38 | 0.61 |
| 1 | 36 | 70 | 850 | 0.14 | 0.91 | 6.16 | 6.61 | 151 | 0.16 | 0.97 | 3476 | 193 | 3.16 | 0.03 |
| 1 | 37 | 70 | 850 | 0.14 | 0.87 | 0.60 | 6.63 | 122 | 0.69 | 0.84 | 3210 |  | 2.79 |  |
| 1 | 38 | 70 | 850 | 0.03 | 0.75 | 8.46 | 6.51 | 150 | 1.14 | 0.77 | 4381 |  | 3.29 |  |
| 1 | 39 | 70 | 850 |  | 0.7 | 5.71 | 6.86 | 124 | 0.99 | 0.78 | 4641 |  | 3.25 |  |
| 1 | 40 | 70 | 850 | 0.12 | 0.76 | 2.88 | 6.5 | 200 | 0.65 | 0.72 | 3569 |  | 2.71 |  |
| 1 | 41 | 100 | 850 | 0.5 | 1.14 | 3.52 | 7.36 | 107 | 0.27 | 0.95 | 1989 | 1763 | 2.27 | 0.88 |
| 1 | 42 | 100 | 850 | 0.25 | 1.06 | 3.31 | 6.67 | 110 | 0.69 | 0.73 | 1699 | 5438 | 1.80 | 1.36 |
| 1 | 43 | 100 | 850 | 0.15 |  | 5.49 | 6.63 | 115 | 0.56 | 0.75 |  | 7958 |  | 1.19 |
| 1 | 44 | 100 | 850 | 0.78 | 1.23 | 5.49 | 6.57 | 127 | 0.37 | 0.66 | 1635 | 1257 | 2.01 | 0.98 |
| 1 | 45 | 100 | 850 | 0.31 | 1.02 | 3.52 | 7.05 | 113 | 0.37 | 0.86 | 2035 | 4664 | 2.08 | 1.45 |
| 2 | 166 | 30 | 400 | 0.04 | 0.73 | 9.41 | 5.97 | 154 | 1.02 | 0.70 | 4602 | 26946 | 3.36 | 1.08 |
| 2 | 167 | 30 | 400 | 0.37 | 0.92 | 3.52 | 6.55 | 120 | 0.05 | 0.76 | 2015 |  | 1.85 |  |
| 2 | 168 | 30 | 400 | 0.21 | 0.94 | 1.83 | 6.33 | 131 | 0.23 | 0.76 | 3227 | 5714 | 3.03 | 1.20 |
| 2 | 169 | 30 | 400 | 0.66 | 0.92 | 4.60 | 4.54 | 0 | 0.07 | 0.58 | 2280 | 1336 | 2.10 | 0.88 |
| 2 | 170 | 30 | 400 | 0.53 | 0.94 | 3.09 | 6.56 | 165 | 0.07 | 0.59 | 2152 |  | 2.02 |  |
| 2 | 171 | 70 | 400 | 0.08 | 0.87 | 7.53 | 6.03 | 184 | 12.12 | 0.81 | 4499 | 22577 | 3.91 | 1.81 |
| 2 | 172 | 70 | 400 | 0.16 | 0.78 | 4.17 | 5.99 | 162 | 1.80 | 1.01 | 4671 |  | 3.64 |  |
| 2 | 173 | 70 | 400 |  | 0.91 | 3.31 | 6 | 174 | 1.59 | 0.94 | 3605 |  | 3.28 |  |
| 2 | 174 | 70 | 400 |  | 0.78 | 2.67 | 5.85 |  | 21.21 | 0.89 | 4930 |  | 3.85 |  |
| 2 | 176 | 100 | 400 |  | 0.61 | 6.61 | 6.47 | 153 | 26.89 | 0.66 | 6752 |  | 4.12 |  |
| 2 | 177 | 100 | 400 |  | 0.53 | 10.62 | 5.93 | 123 | 2.16 | 1.10 | 1456 |  | 0.77 |  |
| 2 | 178 | 100 | 400 |  | 0.52 | 14.94 | 6.48 | 145 | 12.24 | 0.76 | 7159 |  | 3.72 |  |
| 2 | 179 | 100 | 400 |  | 0.61 | 3.09 | 6.5 | 117 | 10.60 | 0.68 | 6888 |  | 4.20 |  |
| 2 | 180 | 100 | 400 |  | 0.55 | 10.13 | 6.45 | 127 | 11.58 | 0.78 | 7368 |  | 4.05 |  |
| 2 | 181 | 30 | 850 | 0.33 | 0.91 | 9.41 | 6.5 | 98 | 0.15 | 0.73 | 1638 | 2357 | 1.49 | 0.78 |
| 2 | 182 | 30 | 850 | 0.24 | 1.18 | 3.31 | 6.3 | 132 | 0.03 | 0.64 | 1201 | 2720 | 1.42 | 0.65 |
| 2 | 183 | 30 | 850 | 0.72 | 1.12 | 2.88 | 6.36 | 126 | 0.04 | 0.78 | 1376 | 1121 | 1.54 | 0.81 |
| 2 | 184 | 30 | 850 | 1.11 | 1.51 | 7.30 | 6.18 | 143 | 0.05 | 0.79 | 1357 | 764 | 2.05 | 0.85 |
| 2 | 185 | 30 | 850 | 0.47 | 1.12 | 2.25 | 6.19 | 131 | 0.05 | 0.75 | 1262 | 1888 | 1.41 | 0.89 |
| 2 | 187 | 70 | 850 |  | 0.57 | 1.42 | 6.2 | 130 | 2.37 | 0.68 | 5913 |  | 3.37 |  |
| 2 | 189 | 70 | 850 |  | 1 | 3.52 | 6.29 | 90 | 0.18 | 0.81 | 2352 |  | 2.35 |  |
| 2 | 190 | 70 | 850 |  | 0.87 | 1.83 | 6.16 | 108 | 0.75 | 0.76 | 3668 |  | 3.19 |  |
| 2 | 191 | 100 | 850 |  | 0.91 | 3.09 | 6.3 | 105 | 0.15 | 0.64 | 3225 |  | 2.93 |  |
| 2 | 192 | 100 | 850 |  | 0.96 | 4.82 | 6.25 | 95 | 0.60 | 0.85 | 3368 |  | 3.23 |  |
| 2 | 193 | 100 | 850 | 0.14 | 1.26 | 1.83 | 6.37 | 99 | 0.10 | 0.76 | 2152 | 11494 | 2.71 | 1.61 |
| 2 | 194 | 100 | 850 | 0.03 | 0.96 | 5.26 | 6.2 | 96 | 0.27 | 0.81 | 3167 |  | 3.04 |  |
| 2 | 195 | 100 | 850 | 0.1 | 1 | 2.67 | 5.96 | 97 | 0.26 | 0.95 | 2966 |  | 2.97 |  |
| 3 | 201 | 30 | 400 | 0.42 | 0.81 | 4.17 | 6.6 | 147 | 0.16 | 0.57 | 4409 | 1727 | 3.57 | 0.73 |
| 3 | 202 | 30 | 400 |  | 0.47 | 6.16 | 6.83 | 144 | 1.70 | 0.58 | 8914 |  | 4.19 |  |
| 3 | 203 | 30 | 400 | 0.38 | 0.86 | 7.07 | 6.32 |  | 0.14 | 0.56 | 2655 | 2233 | 2.28 | 0.85 |
| 3 | 204 | 30 | 400 | 0.34 | 0.85 | 3.73 | 6.09 | 189 | 0.21 | 0.57 | 2364 |  | 2.01 |  |
| 3 | 205 | 30 | 400 | 0.4 | 1.06 | 2.04 | 6.52 | 132 | 0.17 | 0.44 | 2370 | 2258 | 2.51 | 0.90 |
| 3 | 196 | 70 | 400 |  | 1.07 | 3.31 | 6.27 | 174 | 1.04 | 0.77 | 3494 |  | 3.74 |  |
| 3 | 197 | 70 | 400 |  | 0.97 | 3.95 | 6.46 | 162 | 1.62 | 0.83 | 3755 |  | 3.64 |  |
| 3 | 198 | 70 | 400 |  | 1.03 | 1.83 | 6.4 | 146 | 1.18 | 1.18 | 4194 |  | 4.32 |  |
| 3 | 199 | 70 | 400 | 0.06 | 0.98 | 1.83 | 6.78 | 167 | 0.41 | 0.74 | 3006 |  | 2.95 |  |
| 3 | 200 | 70 | 400 |  | 0.65 | 2.67 | 6.86 | 146 | 0.76 | 0.99 | 6404 |  | 4.16 |  |
| 3 | 206 | 100 | 400 |  | 0.7 | 0.20 | 6.52 | 152 | 1.53 | 0.84 | 4713 |  | 3.30 |  |
| 3 | 207 | 100 | 400 |  | 0.84 | 2.25 | 6.7 | 149 | 1.22 | 0.84 | 4300 |  | 3.61 |  |
| 3 | 208 | 100 | 400 |  | 0.46 | 6.84 | 6.57 | 184 | 10.01 | 0.61 | 8186 |  | 3.77 |  |
| 3 | 209 | 100 | 400 |  | 0.34 | 10.13 | 6.6 | 191 | 16.11 | 0.60 | 11038 |  | 3.75 |  |
| 3 | 210 | 100 | 400 |  | 0.54 | 7.30 | 6.68 | 170 | 2.68 | 0.55 | 7928 |  | 4.28 |  |
| 3 | 211 | 30 | 850 | 0.27 | 1.32 | 3.73 | 6.65 | 188 | 0.15 | 0.46 | 1484 | 3144 | 1.96 | 0.85 |
| 3 | 212 | 30 | 850 | 0.64 | 1.23 | 1.21 | 6.73 | 181 | 0.21 | 0.48 | 1306 | 1090 | 1.61 | 0.70 |
| 3 | 213 | 30 | 850 | 0.58 | 1.49 | 2.67 | 6.72 | 190 | 0.13 | 0.70 | 788 | 1268 | 1.17 | 0.74 |
| 3 | 214 | 30 | 850 | 0.29 | 1.28 | 6.16 | 6.75 | 188 | 0.12 | 0.75 | 1274 | 2698 | 1.63 | 0.78 |
| 3 | 215 | 30 | 850 | 0.52 | 1.12 | 5.04 | 6.2 | 192 | 0.13 | 0.78 | 1289 | 1515 | 1.44 | 0.79 |
| 3 | 216 | 70 | 850 | 0.19 | 0.81 | 2.88 | 6.22 | 178 | 0.74 | 0.65 | 3985 | 7736 | 3.23 | 1.47 |
| 3 | 217 | 70 | 850 | 0.06 | 1.11 | 4.38 | 6.74 | 164 | 1.35 | 0.59 | 2815 |  | 3.12 |  |
| 3 | 218 | 70 | 850 | 0 | 0.81 | 7.30 | 6.67 | 152 | 8.56 | 0.50 | 4300 |  | 3.48 |  |
| 3 | 219 | 70 | 850 | 0.14 | 1.18 | 1.21 | 6.03 |  | 0.66 | 0.74 | 2637 | 10736 | 3.11 | 1.50 |
| 3 | 220 | 70 | 850 | 0.1 | 0.94 | 4.82 | 6.22 | 203 | 1.98 | 0.54 | 3008 |  | 2.83 |  |
| 3 | 223 | 100 | 850 | 0.1 | 1.02 | 0.60 | 6.59 | 85 | 0.39 | 0.66 | 2808 | 13769 | 2.86 | 1.38 |
| 3 | 224 | 100 | 850 | 0.09 | 0.97 | 3.09 | 6.44 | 105 | 1.13 | 0.98 | 3055 |  | 2.96 |  |
| 3 | 225 | 100 | 850 | 0.08 | 1.05 | 7.30 | 6.13 | 173 | 0.82 | 1.07 | 2605 |  | 2.74 |  |
